# The underpinning of meaningful activities by brain correlates: a systematic review

**DOI:** 10.3389/fpsyg.2023.1136754

**Published:** 2023-04-26

**Authors:** Ellen Cruyt, Patricia De Vriendt, Nele De Geyter, Janne Van Leirsberghe, Patrick Santens, Stijn De Baets, Miet De Letter, Peter Vlerick, Patrick Calders, Robby De Pauw, Kristine Oostra, Dominique Van de Velde

**Affiliations:** ^1^Department of Rehabilitation Sciences, Faculty of Medicine and Health Sciences, Occupational Therapy Research Group, Physiotherapy and Speech-Language Pathology/Audiology, Ghent University, Ghent, Belgium; ^2^Department of Occupational Therapy, Artevelde University of Applied Sciences, Ghent, Belgium; ^3^Mental Health Research Group, Vrije Universiteit Brussel, Brussels, Belgium; ^4^Frailty in Ageing Research Group, Vrije Universiteit Brussel, Brussels, Belgium; ^5^Department of Neurology, Ghent University Hospital, Ghent, Belgium; ^6^Department of Work, Organization and Society, Faculty of Psychology and Educational Sciences, Ghent University, Ghent, Belgium; ^7^Lifestyle and Chronic Diseases, Department of Epidemiology and Public Health, Sciensano, Brussels, Belgium; ^8^Department of Physical and Rehabilitation Medicine, Ghent University Hospital, Ghent, Belgium

**Keywords:** occupations, meaning, neurophysiology, brain imaging techniques, activities, brain processes, systematic review

## Abstract

**Introduction:**

Engaging in meaningful activities contributes to health and wellbeing. Research identifies meaningfulness by analysing retrospective and subjective data such as personal experiences in activities. Objectively measuring meaningful activities by registering the brain (fNIRS, EEG, PET, fMRI) remains poorly investigated.

**Methods:**

A systematic review using PubMed, Web of Science, CINAHL, and Cochrane Library.

**Findings:**

Thirty-one studies investigating the correlations between daily activities in adults, their degree of meaningfulness for the participant, and the brain areas involved, were identified. The activities could be classified according to the degree of meaningfulness, using the attributes of meaningfulness described in the literature. Eleven study activities contained all attributes, which means that these can be assumed to be meaningful for the participant. Brain areas involved in these activities were generally related to emotional and affective processing, motivation, and reward.

**Conclusion:**

Although it is demonstrated that neural correlates of meaningful activities can be measured objectively by neurophysiological registration techniques, “meaning” as such has not yet been investigated explicitly. Further neurophysiological research for objective monitoring of meaningful activities is recommended.

## 1. Introduction

The need to engage in meaningful activities has already been described a long time ago in occupational science as a basic human drive (Yerxa, 1990). Performing meaningful activities protects against the risk of all-cause mortality and has a positive effect on health (Persson and Jonsson, 2009; Ratra and Singh, 2022), wellbeing (White et al., 2013), and quality of life (Morley et al., 2014). “Meaningful” as defined by Townsend and Polatajko (2007) is “what a person creates for oneself in one's mind that explains experiences and, in turn, motivates and spurs to create new experiences.” People automatically attribute meaning to the experience of their optimal engagement in activities (Steger, 2013). Such an attribution refers to a cognitive component of meaningfulness (Heine et al., 2006), which further provides a basis for the identification of individual goals and purposes and in a broader sense a mission that is personally or culturally important (Kielhofner, 2009). This sense of purpose underlies the motivational component of the concept of meaning. Motivation is indeed a necessary component in addition to the cognitive aspect of meaning in order to engage in meaningful activities (Steger et al., 2008). Since motivation is at least in part dependent on reward mechanisms, an activity can also be defined as meaningful to the degree that it can trigger an intrinsic reward (Bracke et al., 2006). Furthermore, meaningful activities are also related to positive emotions (e.g., happiness) or positive affect such as laughter (Jang and Chiriboga, 2011; Kim et al., 2015).

To date, evidence exists that every activity, ranging from basic activities of daily living, such as bathing and clothing, to higher levels of daily activities, such as having a job, has the potential to obtain a degree of meaningfulness (Kreiss and Schnell, 2022). In other words, any domain in the “activity and participation” component of the International Classification of Functioning, Disability, and Health (ICF) (World Health Organization, 2001) can be meaningful to a person (Van de Velde et al., 2016).

However, the complexity of the concept of meaning resulted in various definitions, found within occupational science, philosophy, natural science, human and social science, and health sciences (Erlandsson et al., 2011; Eakman, 2012). For example, within the social sciences, meaning is constructed on a social basis, so the social environment plays an important role. Within occupational science, an academic discipline dealing with the concept of human occupation (also defined as meaningful activities), this construct of meaning is thoroughly rooted. The person experiences meaning through the optimal transactional relation between person, activity, and environment (Dickie et al., 2006). Meaningfulness may vary considerably depending on the activity considered, the individual, and the environment. This can result in fragmented literature causing confusion regarding how meaning-making occurs. The lack of consistency has hindered theoretical development of meaning (Eakman, 2011). Hammell (2004) addressed this concern, advocating for a conceptualisation of occupation, dividing the concept into dimensions of meaning that capture research findings from participants who have experienced occupational disruption. He reflected on the findings of Wilcock et al. (1998) and Rebeiro (2001) where doing, being, belonging, and becoming were the agreed dimensions. “Doing” referred to the purposeful and goal-oriented activities. “Being” stands for reflecting, being introspective. “Belonging” described the connection with the social environment. “Becoming” means to envision future selves.

Other attempts were made to capture the nature of meaning in the experience of activity. A conceptualisation study based on primary studies in occupational science resulted in an occupational meaning system with interconnected themes and forms of meaning associated with occupations such as; identity, belonging, enjoyment, purposes, autonomy etc. (Eakman et al., 2018). Roberts and Bannigan (2018) conducted a meta-analysis and found that fulfilment, identity, restoration, social, cultural, and intergenerational connection were common dimensions of personal meaning. A more recent concept analysis of meaningful activities identified five attributes of meaningfulness: (a) enjoyable; (b) suited to the individual's skills, abilities and preferences; (c) related to personally relevant goals; (d) engaging; and (e) related to an aspect of identity (Tierney and Beattie, 2020). This study included research of adults in general and elderly with dementia and translated these attributes to people with dementia since research has shown that attributes of meaning in adults have a striking resemblance to findings of people with dementia (Phinney et al., 2007). Research even suggests that activity preferences do not change following diagnosis of dementia including meaningful activities. These attributes can be linked with the flow theory of Csikszentmihalyi (1997) where the individual's capabilities and degree of challenge in the activity is so closely aligned that the experience is intensely enjoyable resulting in a natural flow state. This flow state may reflect the cognitive and emotional meaning the activity has for the individual because the activity can be achieved as it is neither too demanding nor too simple.

Another level of evidence which contributes to the explanation of meaning is based on frameworks and conceptual models which incorporate components which are essential in the process of meaning-making in an activity (Eschenfelder, 2005). For instance, the theoretical framework “Occupational Form, Occupational Performance” showed that meaningfulness of an occupation refers to an individual's interpretation of an occupational form (Nelson, 1988). The occupational form in this framework is described as the preexisting structure that evokes, guides, or structures activity performance. In a recent conceptual model; the “Value and Meaning in Occupation model” (ValMO), meaning is described as a thorough interaction of person-task-environment triad and started from the values of occupations. In this model, an additional exploration of the type of meaning is given. They operationalized meaning as the occupational value which in turn is composed of three dimensions: concrete value, symbolic value, and self-reward value. The self-reward value or dimension comprises experiences of pleasure and enjoyment where the person forgets the time and is in a state of flow, as highlighted by Kielhofner (2002) and Hammell (2004). Another example is given in the Model of Human Occupation (MOHO). In this model, “volition” is embedded as a human subsystem that is important in meaning-making through personal roles and the interaction of the mind, brain, and body (Kielhofner, 2002).

The list of concepts or models are iterative, each with their own theoretical basis. Nonetheless, current studies identifying aspects and similarities in the meaningfulness of activities, mainly rely on retrospective data based on the measurement of the personal experience(s) of meaningfulness in activities. These were mostly captured by means of qualitative methods in which in-depth interviews or self-reported measures were applied (Roberts and Bannigan, 2018). Given the strengths and weaknesses of these subjective methods, the question arises whether the “meaning aspects” of daily life activities can be captured through and/or should be complemented by other, more objective methods.

Ikiugu et al. (2016) did an attempt to explain objectively the contribution of meaningful activities to health and wellbeing by testing the hypothesis of Gutman and Schindler (2007) that meaningful occupations activate the reward (dopaminergic) neural pathways involving the frontal cortex, the ventral tegmental area, nucleus accumbens, anterior cingulate cortex, amygdala, and the hippocampal formation. They used functional magnetic resonance imaging (fMRI) but failed to confirm this hypothesis.

Over the last two decades, the advent of methods for objectively evaluating activities by neurophysiological measurements has resulted in new clinical applications and modalities (Orrison et al., 2017). Well-established neuroimaging techniques such as fMRI, functional near-infrared spectroscopy (fNIRS), brain positron emission tomography (PET), and electrophysiological techniques based on electroencephalography (EEG), have made it possible to non-invasively measure human brain functions while engaging in an activity (Raichle, 2000). This has become one of the major quests in contemporary neuroscience.

It has to be acknowledged that the necessity to standardise tasks and paradigms for their use in neuroimaging or electrophysiological studies, often prevents the interpretation of results in terms of meaningfulness as experienced by the participant (Caspers et al., 2010).

Indirect factors related to meaningfulness have already been studied, but only in resting-state brain studies. Wellbeing can be correlated to activity in the prefrontal cortex, insula, and anterior cingulate cortex (Rickard and Vella-Brodrick, 2014). Kong et al. (2016) found, in a resting-state fMRI study on the correlates of social wellbeing, that the pursuit of meaningfulness and engagement mediated the effect of the fractional amplitude of low-frequency fluctuations (fALFF) in the right posterior superior temporal gyrus on social wellbeing, whereas the pursuit of engagement mediated the effect of the fALFF in the right thalamus on social wellbeing. Chen et al. (2015) reported an increase of coherent connections in resting-state EEG that were associated with higher levels of happiness. The review of Gutman and Schindler (2007) indicated that purposeful and meaningful activities contribute to the enhancement of wellbeing and to positive health outcomes by the reduction of stress and the slowing of cognitive decline.

However, the neural correlates of meaningfulness while performing activities (and not only during resting-state), remain unclear. In this paper, we systematically reviewed the literature on neurophysiological investigations that investigated daily meaningful activities with the aim of identifying the neural correlates of meaningfulness. We focused on studies investigating brain activation during the performance of meaningful activities by means of EEG, fNIRS, fMRI, and PET. We are aware that a systematic review is retrospective in nature. Therefore, we aim to analyze the data based on the current state of knowledge.

The main research question is therefore: “What are the neural correlates of meaningful activities as measured by EEG, fNIRS, fMRI, and PET in healthy adults?”

## 2. Methods

This systematic review was performed following the guidelines of the Preferred Reporting Items for Systematic Review and Meta-Analysis (PRISMA) statement, version 2020 (Page et al., 2020).

### 2.1. Information sources and search strategy

To decrease the risk of bias, three independent researchers (EC, JVL, NDG) conducted the search. They were supervised by the review team of senior researchers with experience in this particular domain and with various backgrounds such as occupational therapy, medicine, biology, speech and language therapy, physiotherapy, and psychology.

The search was conducted in October, 2022. The databases PubMed, Web of Science, CINAHL, and Cochrane Library were used. A preliminary search was conducted to determine the free terms and Medical Subject Headings (MeSH) providing the highest number of relevant results. An overview of the search strings in PubMed can be found in Supplementary Table S1. Key terms were combined into search phrases using Boolean operators.

### 2.2. Eligibility criteria

Publications were included if the study (1) investigated the performance of activities of daily life, (2) was published between January 2000 and October 2022, (3) was written in English, (4) included healthy persons or persons with a non-neurological chronic disease, with a mean age 18 years and older, (5) used fMRI, EEG, fNIRS or PET to investigate brain activation elicited by the task and the results reported the activated brain areas, (6) was peer reviewed, and (7) included the name of the activity in the abstract and (8) the activity had the potential to be meaningful for the participants. The latter inclusion criterion was operationalized as followed:

To decide whenever a study activity was meaningful for the participant, a framework was used with attributes of meaning. After a comprehensive literature search, the authors agreed on using the concept-analysis of Tierney and Beattie (2020) which includes five attributes to make an activity meaningful. We chose these attributes because they are intuitive, clear, and can be efficiently applicate in the articles without asking the participants. These five attributes resulted from literature relevant to adults more generally, but with consideration for application to people with dementia. Since it is acknowledged that meaning in activities in people with dementia bears a striking resemblance to findings from healthy adults (Phinney et al., 2007), these five attributes were used in this study to check whether the studied activities involved meaning for the participants.

Firstly, the activity had at least two of the five attributes of meaning. These attributes were: (a) enjoyable; (b) suited to the individual's skills, abilities and preferences; (c) related to personally relevant goals; (d) engaging; and (e) related to an aspect of identity. Secondly, the activity should be described as a category in the ICF under the component ‘activity and participation' or otherwise be linked to an existing category by using the linking rules in order to ensure that the assignment to the categories was consistent (Supplementary Table S2) (Cieza et al., 2019).

Publications were excluded if (1) they were animal studies; (2) the studies involved drugs or resting-state brain scans; (3) the participants were in an acute phase of (chronic) disease or had neurologic deficits (e.g., stroke, dementia, Alzheimer's) and/or psychiatric disorders; or (4) the studied activity elicited negative responses in the brain such as workload and stress; or (5) the study was about the evaluation of an intervention (pre-and post-measures).

### 2.3. Selection process

After the initial literature search, papers were screened in three steps, (1) duplicates were screened using the reference management system Endnote X9, (2) the three researchers EC, JVL, NDG independently applied the inclusion criteria to the title and abstract, (3) full-text versions of the retained articles were retrieved and reviewed against the inclusion criteria. In this step, the meaning type (following Tierney et al.) and activity type (following ICF linking rules) were also screened independently by the three researchers. If there were any disagreements of inclusion/exclusion, the three reviewers organised a consensus meeting to decide. After the second and third phase, Cohen's kappa statistics were calculated. Each article included in the review underwent a methodological quality appraisal using the Mixed Method Appraisal Tool (MMAT) (Supplementary Table S3) (Hong et al., 2018). The risk of bias was evaluated for each article using the Critical Appraisals Tool from Joanna Briggs Institute (Supplementary Table S4) (Munn et al., 2020). These tools assure that every included article, whether it is qualitative, quantitative, or mixed design, can be evaluated using the same criteria concerning the level of evidence and risk of bias.

### 2.4. Synthesis of the results

Data were extracted and information was collated in an evidence table (Supplementary Table 1) organised as follows: (a) authors, year, country, (b) neurophysiological measurement technique, (c) study design, level of evidence, and risk of bias, (f) participants, (g) main activity, (h) the domain of the ICF the activity belongs, (i) MMAT score, (j) aim of the study (k) main results concerning the study subject of the systematic review. This last named item was retrieved by searching for each study in the neuroscience literature about the activated brain regions and their functions. We compared these functions to possible characteristics of meaning in an activity. The studies were grouped in the table based on the measurement techniques.

### 2.5. Thematic analysis to define the level of meaningfulness in the studied activities

Thematic analysis discussed the degree of meaningfulness for the participant in the study activity. Based on the attributes of Tierney and Beattie (2020), the studies were grouped together.

The studies with the most attributes of meaningfulness were discussed per measurement technique and specific correlations of brain activation with possible meaningful elements were extracted. The same method was used as for retrieving the main results of each study as mentioned here above (item k). The main results were linked to functions that can be related to the meaning aspect as stated in the introduction.

## 3. Results

### 3.1. Study selection

**Figure 1:**
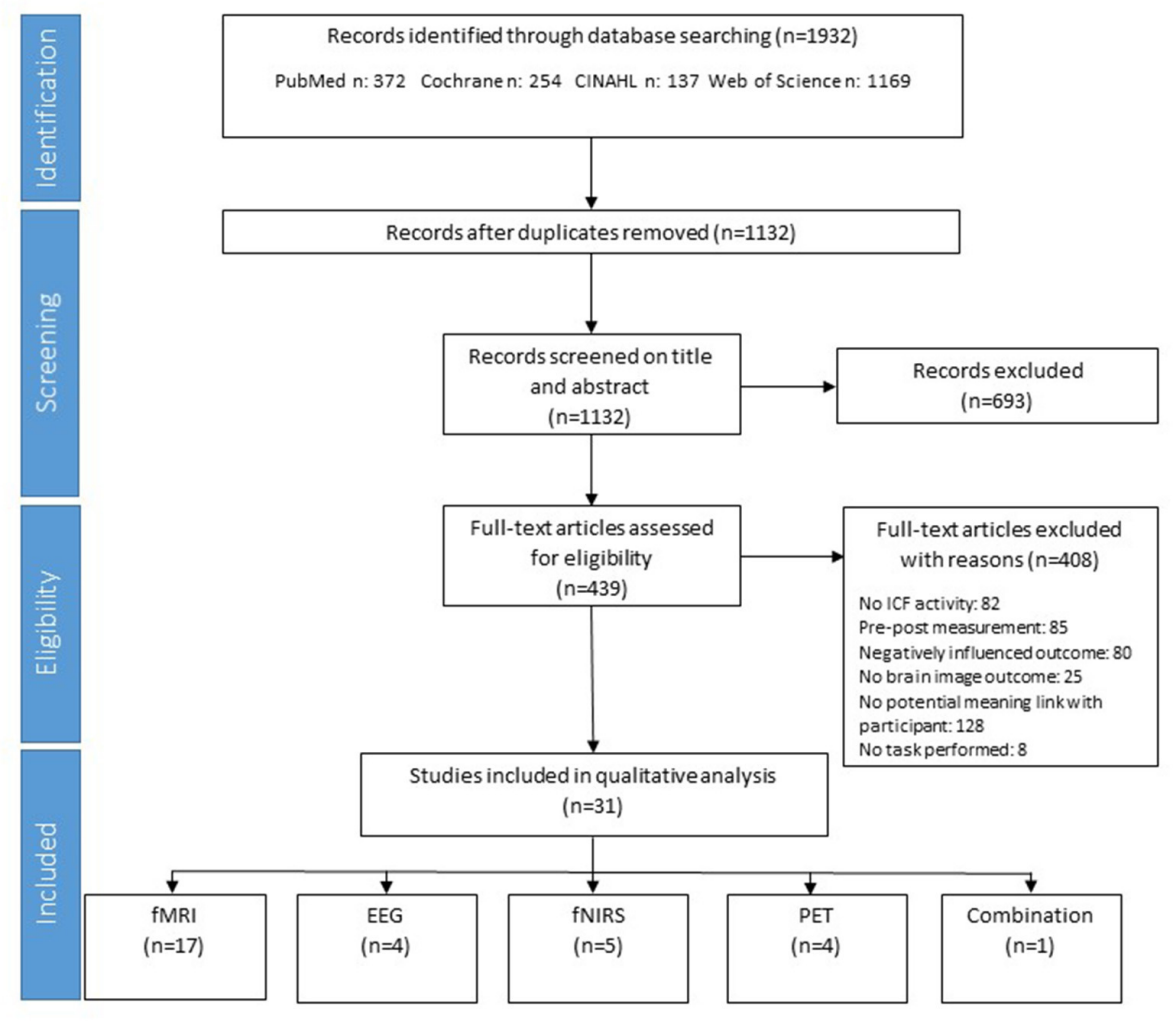
Prisma flowchart of the strategy.

The flowchart according to the PRISMA statement displays the literature retrieval process (Figure 1) (Wilcock et al., 1998). Articles were included for the next step if two or three researchers agreed to include the article based on title and abstract (mean kappa = 0.64: substantial agreement). In total, 439 articles were read in full text (mean kappa = 0.75: substantial agreement).

### 3.2. Study characteristics

In what follows, a description of the included articles is given. To maintain the readability, the references were included in Supplementary Table S5. Supplementary Table S6 showed the degree of meaningfulness based on the attributes of Tierney and Beattie (2020).

#### 3.2.1. Description of the studies

The most common reasons for exclusion were: (1) the activity did not contain at least two attributes of meaningfulness for the participant (31%), (2) the measurement happened in a pre or post measurement (21%), and (3) the activity did not fit in the activity section of the ICF (20%).

The 31 selected studies (Supplementary Table 1) were published between 2001 and 2022. Studies were conducted in Canada (*n* = 5), the USA (*n* = 4), China (*n* = 4), Germany (*n* = 3), Sweden (*n* = 3), Japan (*n* = 2), Portugal (*n* = 1), France (*n* = 2), Italy (*n* = 2), Austria (*n* = 1), Ireland (*n* = 1), Brazil (*n* = 1), Korea (*n* = 1), and UK (*n* = 1).

#### 3.2.2. Measurement techniques

The most used measurement technique in the included articles was fMRI (*n* = 17). Five studies used fNIRS, four studies used EEG, and four studies worked with PET scans. One study used the combination of PET and fMRI on separate moments but while performing the same activity.

#### 3.2.3. Aim of the studies

Sixteen studies aimed to find the neural network or the selected regions of interest during a specific activity. Seven studies focused on neural mechanisms to detect emotional responses related to the given activity. Two studies aimed to detect the motivational region in the brain. One study detected the preferences of the participants for an activity through brain imaging. Four studies evaluated the feasibility of the measurement technique and one study attempted to define the reward area in the brain.

#### 3.2.4. Population

Overall, the majority of the studies (*n* = 21) used a mixed group of both women and men with four studies balanced for gender. Nineteen studies specified right-handed adults, and six studies specified healthy students. No studies were found with adults suffering from a non-neurological chronic disease. In total, 625 participants were included in the studies. Across most of the studies (*n* = 17), the mean age varied between 20 and 30 years.

#### 3.2.5. Type of activities

A variety of activities was used to study the human brain. In seventeen studies, the main activities were passive, including nine involving listening to music. Four studies concerned recalling a memory, and five focused on reflecting while watching a movie.

Fifteen studies used active participation of subjects by performance of a physical activity, e.g., dancing. There was only one study with the activity being performed outside the lab, using table tennis, piano play and daily work activities in the ecological setting.

Based on the domains for Activities and Participation in the ICF, 19 studies were situated in the D1 *learning and applying knowledge*. Six studies in D9 *community, social and civic life*, four in D4 *mobility*, three studies in D2 *general tasks and demands*, and one in D5 *self-care*.

#### 3.2.6. The degree of meaningfulness in the activities

The included articles contained an activity which had two or more attributes based on the concept analysis of Tierney and Beattie (Tierney and Beattie, 2020) that define an activity as meaningful. However, a difference in the number of attributes can be observed (Supplementary Table S6).

In two publications, only two attributes fitted the activity (Walter et al., 2001; Schweizer et al., 2013). Both activities suited to the individual skills and were engaging for the participants.

Three studies concerned activities that contained three different attributes (Small et al., 2001; Karmonik et al., 2016; Pan et al., 2021), sixteen with four attributes (Bengtsson and Ullén, 2006; Spiers and Maguire, 2006; Mathiak et al., 2011; Wan et al., 2011; Klasen et al., 2012; Matsunaga et al., 2014; Chen et al., 2017; Gatti et al., 2017; Verdiere et al., 2018; Ara and Marco-Pallares, 2020; Jensen et al., 2020; Shane et al., 2020; Chang et al., 2021; Marion et al., 2021; Zhou et al., 2022) wherein one study did not relate the activity to the identity of the participant (Matsunaga et al., 2014) and 14 studies did not include personally relevant goals (Raichle, 2000; Walter et al., 2001; Gutman and Schindler, 2007; Caspers et al., 2010; Schweizer et al., 2013; Rickard and Vella-Brodrick, 2014; Chen et al., 2015; Ikiugu et al., 2016; Kong et al., 2016; Orrison et al., 2017; Hong et al., 2018; Cieza et al., 2019; Munn et al., 2020; Page et al., 2020).

In 11 studies the activities contained all of the five attributes of meaningful activities (Blood and Zatorre, 2001; Perreau-Linck et al., 2007; Pereira et al., 2011; Moghimi et al., 2012; Pinho et al., 2014; Balardin et al., 2017; Boissoneault et al., 2018; Chabin et al., 2020; Jin et al., 2020, 2021; Lee and Reeve, 2020; Talami et al., 2020). Only these studies contained the attribute of “related to personally relevant goals.” Although the researchers determined the “general” activity, there was room left for the participant to fulfil, pursue and achieve personally relevant goals. The participants can therefore have a sense of self-worth, competence, control, satisfaction, etc. For example, ballroom dancers completed their favourite dance on a preferred tapping tempo.

### 3.3. Results of the studies involving a potential meaningful activity

A closer look into the results of the latter 11 studies with activities containing the five attributes leading to a meaningful activity yields a preliminary idea about brain regions involved in the performance of a meaningful activity.

It should be acknowledged that condensing brain functions in specific brain areas is usually a faulty approach, since brain functions are supported by network activities of communicating groups of neurons in various cortical and subcortical regions of the brain. Therefore, the results of the studies in this paper are most probably a first and incomplete approach as we can only report the areas that are possibly related to meaningfulness highlighted in the literature.

#### 3.3.1. PET

An activation of the anterior cingulate cortex was found both in the study of Blood and Zatorre (2001) while listening to self-selected pleasant music, and Perreau-Linck et al. (2007) when recalling a happy memory. This brain area is known to be involved in the processing of affect, attention, and motivation (Bush et al., 2000). It is an integral part of the larger limbic system, which harbours a number of cortical and subcortical areas involved in emotion formation and processing, learning, and memory (Stanislav et al., 2013). Consequently, the cingulate cortex is expected to have a prominent role in linking motivational outcomes to behaviour (e.g., a certain activity induced a positive emotional response, which results in learning). It has strong reciprocal connections to the orbitofrontal cortex, basal ganglia, insula, and frontal lobe. Likewise, in the study of Blood and Zatorre (2001) an additional activation of the medial prefrontal cortex, which contributes to goal achievement and is a part of the brain reward circuitry (Tzschentke, 2000; Matsumoto and Tanaka, 2004), was described. Furthermore, the blood flow increased in the ventral striatum, which is also involved in signalling the presence of/expectation of reward (Knutson et al., 2001; Eldar et al., 2016). Finally, there was an activation of the orbitofrontal cortex which plays a key role in emotional control, by representing the reward value of the goals for action (Rolls, 2019).

#### 3.3.2. fNIRS

Both fNIRS studies with a potential meaningful activity (listening to self-selected musical pieces and the performance of four daily activities) for the participants found activation in the prefrontal cortex (Moghimi et al., 2012; Balardin et al., 2017). This part of the cortex plans complex cognitive behaviour, codes personality expressions, decision making, and moderates social behaviour. It also represents goals and the means to achieve them (Miller and Cohen, 2001). Because the resolution of fNIRS is limited, this is rather global non-specific activation of the prefrontal cortex.

#### 3.3.3. EEG

Increased theta activity was found in the prefrontal and orbitofrontal cortex in the study of Chabin et al. (2020) while listening to favourite pleasurable chill-inducing musical excerpts. Theta activity is important for processing information, formation of memories, internal focus, spiritual awareness, meditation, and affective processing (Aftanas and Golocheikine, 2001). Activation in theta activity related to music appreciation was also found in the temporal lobe which, besides its role in the processing of musical stimuli, plays a role in processing affect/emotions (Monti and Meletti, 2015). The study of Jin et al. (2021), involving dancers who melodically recalled their favourite music pieces and tapping to the tempo, found higher low-beta and high-beta power especially in the occipital lobe. Low-beta is associated with active concentration and high-beta was reported to be involved in reward signalling (HajiHosseini and Holroyd, 2015).

#### 3.3.4. fMRI

Both the superior and inferior frontal gyrus were activated in the study of Boissoneault et al. (2018) when recalling a happy experience. These were suspected to correlate with self-awareness and laughter (superior) and the ability to live sociably and communicate with others (inferior) (Vaca et al., 2011). Greater functional connectivity was found in the cerebellum, similar to the study of Talami et al. (2020) where participants viewed self-selected funny movies. The role of the cerebellum in cognitive and emotional regulation is increasingly recognised, and since there is a high degree of connectivity with the frontal lobes, it is most probably part of a network sustaining emotional processing and its role in complex behaviour (Wolf et al., 2009). In three study activities (listening to familiar music, playing piano, and remembering intrinsic motivation memories) the anterior cingulate cortex was activated (Pereira et al., 2011; Pinho et al., 2014; Lee and Reeve, 2020). The dorsolateral prefrontal cortex, which is the highest cortical area involved in motor planning, organisation and regulation, was activated in the piano study of Pinho et al. (2014) and Hale and Fiorello (2017). The amygdala (involved in tying emotional meaning to memories, reward processing, and decision-making), nucleus accumbens (plays a role in the positive experiences such as desire, motivation, passion, and satisfaction), thalamus (plays a significant role in motor activity, emotion, memory, arousal, and other sensorimotor association functions), and basal ganglia (role in reward and reinforcement, addictive behaviours, and habit formation), were increased in blood oxygen level in the movie studies of Talami et al. (2020) and Cardinal et al. (2003).

The study of Lee and Reeve (2020) found activation in the ventromedial prefrontal cortex during the recall of intrinsically-motivating memories. This region plays a role in the inhibition of emotional responses, in the process of moral decision-making, and self-control (Hiser and Koenigs, 2018). This was the only study that used the word *personal meaning* but with the nuance that it refers to self-endorsement.

## 4. Discussion

This systematic review aimed to identify, document, and evaluate the scientific literature concerning the relation between brain correlates and meaningful activities in adults without neurologic conditions. Thirty-one studies were included which indeed represent a body of knowledge about the relation between meaningful activities and observable activity in the brain. However, none of the studies explicitly searched for the meaningfulness aspect of the activity.

Most studies investigated the activities in laboratory settings and fitted in the first activity-domain of the ICF *learning and applying knowledge*, involving more basic and functional activities such as listening, watching, etc. This is not surprising taking into account that the majority of the included studies used fMRI scanners, which are known for their limitations such as the restricted space, the necessity for participants to remain supine during scanning, and the controlled laboratory environment in which the scans are typically conducted. This restricts the possible activities of the entire activity repertoire of the participant. Researchers are more inclined to use standardised, functional activities that trigger a certain part of the brain. The studies demonstrated that activities of daily living are difficult to perform in a lab or clinic due to these constraints (Okamoto et al., 2004; Pinti et al., 2015). Meaningful situations are also hard to create successfully in the lab because meaningfulness is embedded in a specific context, that cannot be recreated in the lab (Huta, 2017). Consequently, significant disagreement can occur between measurements taken in everyday life and in lab conditions (Kvavilashvili and Ellis, 2004). Therefore, and in order to be ecologically valid, meaningful activities should take place in an environment that is adapted to the person's needs and preferences (National Institute for Health and Care Excellence, 2013). The fNIRS is used to measure the cognitive ability of less standardised activities and can, as opposed to fMRI, be used in more realistic environments due to the wireless conditions (Arenth et al., 2007). Wireless fNIRS and EEG research enable functional examination of cortical activity during everyday activities and may extend the potential for implementing in research on meaningful activities (Okamoto et al., 2004). Nevertheless, to overcome the issue with space restrictions and the non-meaningful lab context, some included studies used the imaginary aspect of an activity. It is proven that thinking of or recalling an activity in the past, activates most of the cortical areas used during the actual performance of the activity (Jeannerod, 2001).

The second-largest group of activities was clustered in the ICF domain D9 *community, social and civic life*. Recreation and leisure are situated herein, and these activities can be closely related to meaningful activities as they involve time that can be filled in voluntarily with activities of choice.

Regarding the degree of meaningfulness in the activities, the study activities can be presumed to be meaningful because they contained two or more attributes that contribute to meaningfulness (Tierney and Beattie, 2020). Moreover, eleven studies included the five attributes related to meaningfulness in an activity. These studies were enriched by the attribute “related to personally relevant goals,” while this was not the case in the other studies. The studies with the highest number of meaningful aspects in the activities required the participant to shape the activity, which means that the participants could choose aspects of the activity related to personal goals or competences which leads to a feeling of accomplishment and control.

However, the actual intensity of engagement or the cognitive processes involved in a certain activity can vary from individual to individual (Salthouse et al., 2002). For one person, participating in the same activity requires productive engagement, but this can be different e.g., for a more experienced individual (Park et al., 2007).

It remains a huge challenge to synthesise all the brain correlates from the included studies. Every technique differed in measures, interpretation, and reporting of the results and all have (dis)advantages. fMRI and PET are superior in measuring deep structures in the brain while fNIRS cannot. The spatial resolution of fMRI is superior to that of all other techniques, while EEG has a superior temporal resolution (Lakshmi et al., 2014). Our research question can be answered by reflecting on the activated brain regions and the electrophysiological alterations in the study activities with the five attributes of meaningfulness. These brain correlates are known to be involved in various related functions of meaning as stated in the introduction, such as affect (anterior cingulate cortex, theta waves, temporal lobe), reward (medial prefrontal cortex, ventral striatum, orbitofrontal cortex, high-beta waves, amygdala, and basal ganglia), motivation (anterior cingulate cortex, nucleus accumbens), emotions [anterior cingulate cortex, orbitofrontal cortex, temporal lobe, inferior frontal gyrus, cerebellum, amygdala, thalamus (ventromedial) prefrontal cortex], goals and means to achieve them (prefrontal cortex), memory (anterior cingulate cortex, theta waves, amygdala, thalamus), and laughter (superior frontal gyrus) (Huta, 2017).

Several reviews in resting-state brain imaging, indicate activation in the prefrontal cortex, insula, temporal gyrus, thalamus, and anterior cingulate cortex which correlate with wellbeing, meaning, and engagement (Gutman and Schindler, 2007; Rickard and Vella-Brodrick, 2014; Kong et al., 2016). We can state that the majority of the included activation studies found one or more of these brain regions activated. All of the publications reporting on activities containing the five attributes of meaningfulness found activation in at least one of them: the PET study of Blood and Zatorre (2001) found activation elicited by activities with a meaningful aspect in the prefrontal cortex, anterior cingulate cortex, and insula; The EEG study of Chabin et al. (2020) found activation in the prefrontal cortex and temporal gyrus; Talami et al. (2020) reported the anterior cingulate cortex and prefrontal cortex during fMRI; Jensen et al. (2020) also in the anterior cingulate cortex and the thalamus during fMRI; the fMRI study of Lee and Reeve (2020) reported the prefrontal and anterior cingulate cortex. In the studies based on four attributes: both the EEG study of Ara and Marco-Pallares (2020) and the fMRI study of Zhou et al. (2022) found activation in the temporal gyrus; Chang et al. (2021) found activation in the prefrontal cortex during fNIRS. We may assume that there is a link between the similar activation of brain areas in activation studies by meaningful activities and the brain areas that are activated under conditions of wellbeing, meaningfulness and engagement. In the studies based on three attributes: Small et al. (2001) found during the PET scan activation in the prefrontal cortex and insula, where sensory experiences are combined in the emotional context; Karmonik et al. (2016) found during the fMRI scan activation in the temporal gyrus, thalamus, and anterior cingulate cortex.

### 4.1. Strengths and limitations

Since the term “meaningful activities” is very broad, it is not possible to label all activities of daily life as terms in the search-string (e.g., sometimes the activity, such as “dancing,” is specifically named in the title and the abstract, but the terms “activity” or “meaningful activity” are not given). The search string was sensitive and a lot of synonyms were used that cover the term meaningful activities. However, it was not possible to name all specific activities in daily life and therefore we may have missed a number of publications. The publications, without the term “meaningful activities” and synonyms but with a stipulation of the activity were also included.

In occupational science, the terms *activity, engagement*, and *meaningful* are important concepts. However, there is disagreement on the unambiguous definition of these terms which makes them more subjective terms (Royeen et al., 2017). Through the search process, it appeared that these terms may have a completely different interpretation in other research fields. Some studies used *activity* and *engagement* to indicate that some brain parts were (co-) active during a task. Note that the term “task” was mostly used as jargon in the brain imaging studies instead of activity. The term task was not included in the search string due to the functional nature of the term. *Meaningful* was used to indicate tasks that were familiar to the participants such as holding a key. These other definitions of the keywords resulted in a high number of irrelevant search results.

The levels of evidence in the included studies were not high, mostly case series were used. This means that there is a need for high-quality RCT's and systematic reviews about this topic.

The aim of this study was to correlate the meaningfulness of activities to neurophysiological alterations in identifiable brain areas. Determining whether a study task was a meaningful activity may seem a subjective decision, especially when this decision happened *post-hoc* without communication with the participants. Since meaning is very broad and very individual, an attempt was made to use recent research with clear and intuitive attributes that can be used to find out whether the activity could be found meaningful by the participants. The addition of the five attributes of Tierney and Beattie (2020) in addition to ICF categories, assured that the evaluation of meaningfulness in an activity happened in a structured and valid way.

## 5. Conclusion and further research

The main finding of this systematic review is that the activities in the included studies were not explicitly designed or tailored as meaningful for the person. The activity was usually studied in terms of a functional research question. However, it can be deduced that these activities could have been meaningful for the participants by categorising these activities in the ICF and by relating attributes of meaningfulness (Tierney and Beattie, 2020). This implies that the general outcomes of these studies do not tell us yet which particular brain area(s) correlate especially with the meaningful aspect of the activity. Moreover, these studies have captured the meaningful aspect of the activity only partially. In other words, there is limited evidence that can support our understanding of meaningful activities and their potential neural correlates. However, we tried to overcome this gap by extracting only the study results that can be linked to meaning, based on a literature search in neuroscience articles. Studies in which the activities can be related to the five attributes of meaning, in which we assume that this must be very meaningful for the participants, were reported on the neural correlates reflecting characteristics such as happiness, reward, emotions, goals, affect etc. These results can give an answer to our research question but should be interpreted at the same time with appropriate caution that meaning in the activities has been attributed based on the theoretical framework of Tierney and Beattie. The answers to the research question should be addressed in this context.

As a systematic review is based on already existing knowledge, and therefore retrospective in nature, we were aware of the fact that identifying neural correlates for specific behavioural constructs are not in line with current developments in the field and that the more recent approach is to acknowledge that the emphasis is on neural networks. Future research should focus on the neural networks. A thematic analysis, based on the current state of knowledge was therefore used to answer the main research question.

Findings from this systematic review demonstrate that brain networks, activated when performing a meaningful activity, have not been explicitly investigated yet. This gap in the literature was also stated in the study of Obrig and Villringer (2003) where they conclude that there is a gap between studies that investigate basic functions, such as motor activities, and studies that investigate higher-level cognitive daily activities. However, researchers so far mainly involved potential meaningful activities in their studies in an indirect or implicit manner and showed that meaningfulness can be correlated to activity in certain brain areas as measured by a variety of techniques.

In addition to looking at active regions in the brain during meaningful activities, it could also be examined whether there is a modification of the electrical activity in those areas that are primarily involved in the task. In addition to the things that are known, for example, that there are spectral changes in the temporal cortex when listening to music, the question can also be asked whether modifications occur at the moment of primary perception.

Future experimental research should explicitly investigate the meaningful activities of participants and implement questionnaires that gauge meaning in activities to ensure that the most meaningful activity is being measured. The research should be supported by a theory or model that links the person with the environment through activity to make sure the meaning aspect is taken into account. Mapping brain correlates accurately during the actual performance of well-defined meaningful activities would not only be more ecologically valid compared to previous studies but may also increase our knowledge of wellbeing in healthy persons. In addition it might even be used as a complementary evaluation tool during rehabilitation in people with brain disorders to optimise performance of meaningful activities (Rolls, 2019). Studies including participants with chronic diseases performing (meaningful) activities were not found in this review, and it would be interesting to focus on them in future research. Similarly, a more objective evaluation of meaningfulness of activities could be highly relevant to improve the quality of life in people with communication disorders and chronic neurodegenerative disorders.

## Data availability statement

The original contributions presented in the study are included in the article/Supplementary material, further inquiries can be directed to the corresponding author.

## Author contributions

EC wrote the manuscript and performed the search. NDG and JVL helped with the search in selecting the articles. SDB checked the included articles. PDV and DVdV supervised the manuscript. MDL, PV, PC, RDP, KO, and PS contributed to manuscript revision and approved the submitted version. All authors contributed to the article and approved the submitted version.
