# Supplementary material for: The underpinning of meaningful activities by brain correlates: a systematic review
Source: Front Psychol. 2023 Apr 26;14:1136754. doi: 10.3389/fpsyg.2023.1136754 (PMC10169732; doi:10.3389/fpsyg.2023.1136754)
Supplement: Supplementary file 1 [file Data_Sheet_1.docx]

Supplementary Material

Table 1. Overview of studies included in the systematic review

| Authors, year, country | Brain registration technique | | Study design  (levels of evidence)  Risk of bias | Participants | | Main activity | ICF | MMAT score | Aim | Outcome measures related to research question |
| --- | --- | --- | --- | --- | --- | --- | --- | --- | --- | --- |
| (Blood & Zatorre, 2001)  Canada | PET | | Quantitative descriptive: case series (4)  Low risk of bias | Students with min. 8 years of music training  5 M 5 F  (20-30y) | | Listening to self-selected pleasant music | d115 | 5 | To examine the neural mechanisms underlying intensely pleasant emotional responses to music | Regional cerebral blood flow increased in left ventral striatum and dorsomedial midbrain, regional cerebral blood flow decreased in right amygdala, left hippocampus/amygdala and ventral medial prefrontal cortex. Regional cerebral blood flow increased with chills intensity in paralimbic regions (bilateral insula, right orbitofrontal cortex), regions associated with arousal (thalamus and anterior cingulate) and motor processes (supplementary motor area and cerebellum). |
| (Small, Zatorre, Dagher, Evans, & Jones-Gotman, 2001)  Canada | PET | | Quantitative descriptive: case series (4)  Low risk of bias | Right-handed chocolate lovers  4 M 5 F | | Eating chocolate | d550 | 5 | To observe recruitment of brain regions depending on whether subjects ate chocolate when they were highly motivated to eat and rated pleasant or ate chocolate despite being satiated. | Highly motivated to eat chocolate: subcallosal region, caudomedial orbitofrontal cortex, insula/operculum, striatum and midbrain. Eating chocolate despite satiated: parahippocampal gyrus, caudolateral orbitofrontal cortex and prefrontal regions. Reward value of food is situated in cortical chemosensory areas, including the insula, caudomedial and caudolateral orbifrontal cortex. Opposite patterns of activity in medial and lateral caudal orbitofrontal cortex. |
| (Matsunaga et al., 2014)  Japan | PET | | Quantitative non-randomized: non-randomized controlled trial (2B)  Low risk of bias | Experiment 2: healthy right-handed students  20 M  (20-30y)  ($\bar{X}$: 22.2y) | | Watching an emotionally neutral film (control) or film featuring people the participants considered attractive | d110 | 4 | To compare the brain responses to positive emotional stimuli of cytosine allele carriers to that of thymine-thymine carriers. | Positive emotion-related brain region such as the medial prefrontal cortex was significantly activated when the cytosine allele carriers watched the positive film compared to the thymine-thymine carriers. |
| (Perreau-Linck et al., 2007)  Canada | PET | | Quantitative descriptive: case series (4)  Low risk of bias | Professional actors  7 M  ($\bar{X}$: 35y) | | Recalling autobiographical memories (happiness, sadness, neutral) | d163 | 5 | To investigate the effect of rapid and sustained changes of emotional state on the trapping of 11C-labelled α-methyl-L-tryptophan (11C-αMtrp) used as a proxy of 5-HT synthesis. | Whole brain analysis revealed positive and negative correlations between experienced levels of emotions and 11C-αMtrp trapping in the right anterior cingulate cortex. |
| (Jin et al., 2020)  China | EEG | | Quantitative non-randomized: non-randomized controlled trial (2B)  Low risk of bias | Right-handed ballroom dancers 10 M 19 F  ($\bar{X}$: 19.8y)  Nondancers (controls)  10 M 20 F  ($\bar{X}$: 23.1y) | | Melodic recall of their favorite music piece and tap to the tempo | d163  d220 | 5 | To investigate if participation in ballroom dancing is associated with elevated cerebral cortical arousal during freely chosen musical recall | Elevated EEG beta power during the musical recall task relative to nondancers, indicating heightened cerebral cortical arousal. Higher low beta and greater high beta in dancers versus nondancers. |
| (Chabin et al., 2020)  France | EEG | | Quantitative descriptive: case series(4)  Low risk of bias | Healthy right-handed adults, sensitive to musical reward and frequently experienced chills by pleasurable music  7 M 11 F  ($\bar{X}$: 39.7y) | | Listen to their favorite pleasurable chill-inducing musical excerpts | d115 | 5 | To show that high-density EEG is able to reveal patterns of cerebral activity | An increase of theta activity in the prefrontal cortex when  arousal and emotional ratings increased (associated with orbitofrontal cortex). Activation patterns of chills: a decreased theta activity in the right central region, which could reflect supplementary motor area activation during chills and may be related to rhythmic anticipation processing, and a decreased theta activity in the right temporal region, which may be related to musical appreciation and could reflect the right superior  temporal gyrus activity. The alpha frontal/prefrontal asymmetry did not reflect the felt emotional pleasure, but the increased frontal beta to alpha ratio (measure of arousal) corresponded to increased emotional ratings. |
| (Ara & Marco-Pallarés, 2020)  Spain | EEG | | Quantitative descriptive: case series(4)  Low risk of bias | Healthy right-handed adults who have similar music preferences  6 M 19 F  ($\bar{X}$: 22.3y) | | Listen to commercially available songs of several music genres | d115 | 5 | To study brain synchronization in the oscillatory band as a function of music-evoked pleasantness | Phase synchronization in the theta band between right temporal and frontal signals increased with the degree of pleasure. |
| (Marion, Di Liberto, Shamma, 2021)  Ireland | EEG | | Quantitative descriptive: case series(4)  Moderate risk of bias | Professional musicians or in training  15 M 6 F  ($\bar{X}$: 25y) | | Listen and imagine monophonic Bach chorals | d115  d163 | 4 | To explore the nature of modulations in imagined music | The note onset encoding was significant at the individual level and was most accurately encoded on central scalp areas. Expectation gain emerged primarily in frontal scalp areas. |
| (Moghimi, Kushki, Power, Guerguerian, & Chau, 2012) Canada | fNIRS | | Quantitative descriptive: case series(4)  Low risk of bias | Adults with an average of 5.5y of music training  5 M 5 F  ($\bar{X}$: 25y) | | Listening to music, with some self-selected musical pieces | d115 | 5 | To exploit hemodynamic activity to automatically decode prefrontal cortical responses to emotionally laden music | Emotional content of music induces differential patterns of activity in the prefrontal cortex. Arousal and valence components of emotion were detected with accuracies higher than 70%.  In both positive and negative rated trails, a decrease in deoxyhemoglobin concentration following hyper-oxygenation is observed. Classification accuracies varies across participants, individual differences in emotional reactivity. |
| (Balardin et al., 2017)  Brazil | fNIRS | | Quantitative descriptive: case-series(4)  Low risk of bias | Ex1: right-handed athlete  1 M  30y  Ex2: right-handed prof. pianist  1 F  51y  Ex3: violin duo  2 M  (41y and 50y)  Ex4: M 26y | | Ex1: table tennis  Ex2: playing piano  Ex3: interaction violin  Ex4: daily work activities | d9201  d9202  d9202  d2302 | 3 | To describe a series of proof-of-concept experiments examining the potential of fNIRS in assessing the neural correlates of cognitive and motor processes in unconstrained environments | Ex1: left motor/premotor regions were more active during unpredictable conditions. Right premotor cortex also more active.  Ex2: right dorsolateral prefrontal cortex was more active in the polyrhythmic than in rhythmically conditions (greater effort is needed)  Ex3: higher synchronization between the brains of the two violinists occurred in parietal and frontal regions, in premotor and somatomotor areas.  Ex4: spectrum suggest periodicity with peaking at 0.002 Hz |
| (Verdiere, Roy, & Dehais, 2018)  France | fNIRS | | Quantitative descriptive: case series(4)  Low risk of bias | Pilots  11 M 1 F  ($\bar{X}$: 24y) | | Manual and automated landing in flight simulator | d4751 | 4 | To assess the potential of connectivity measures to classify two different levels of task engagement with fNIRS under relatively ecological settings. To assess the potential of connectivity measures to better characterize engagement than classical measures. | Connectivity features performed significantly better than the classical concentration metrics with a higher accuracy for the wavelet coherence. All connectivity measures allowed an efficient classification when computed over HbO signals. |
| (Chang, Seccia and Bruce, 2021)  USA | fNIRS | | Quantitative descriptive: case series(4)  Low risk of bias | Healthy adults who followed the graduate-level Digital Video course  2 M 6 F  ($\bar{X}$: 27y) | | Creating a video using IMovie | d197 | 4 | To explore the neurocognitive underpinnings of a Digital Video task. | Results were measured in the prefrontal cortex. A significance difference between hemodynamic responses (HR) were found for Audio, Visual, and Text. HR processing visuals were significantly different from processing texts for each participants. A significant difference was also found in the HR in the tasks Reviewing, Selection/sequencing, Trimming, Special effect, Transition, Text generation. |
| (Pan, Cheng, & Hu, 2021)  China | fNIRS | | Quantitative descriptive: case series(4)  Low risk of bias | Right-handed university students familiar with ancient poems  60 F  ($\bar{X}$: 21.6y) | | Mastering two Chinese ancient poems (cooperatively or independently) | d163  d166 | 4 | To explore the neural mechanisms of learning through cooperation | Significant within-group neural synchronization in the left superior temporal cortex, supramarginal gyrus, and postcentral gyrus during cooperative learning compared to independent learning. |
| (Boissoneault, Sevel, Robinson, & Staud, 2018)  USA | fMRI | | Quantitative descriptive study: case series(4)  Low risk of bias | Healthy adults  7 M 10 F  ($\bar{X}$: 22.4y) | | Recall a fatigue and happy experience | d163 | 4 | To study fatigue without comorbidities, brain activity was studied during recall of a fatiguing event | Only the control task ‘recall happy experience’ was taken for the results; greater functional connectivity between parahippocampal gyrus and a cluster including right supplemental motor area, right superior frontal gyrus, left supplemental motor area was associated with greater happiness ratings. Greater functional connectivity between inferior frontal gyrus and a cluster including right fusiform gyrus, right cerebellum, right lingual gyrus was positive associated with happiness ratings. |
| (Spiers & Maguire, 2006)  UK | fMRI | | Quantitative descriptive study: case series(4)  Low risk of bias | Taxi drivers  20 M  ($\bar{X}$: 49.8y) | | ‘The Getaway’ game, driving a virtual cab with customers in London | d4758 | 4 | To have insight into the content of spontaneous mentalizing events and identify the brain regions that underlie them | Increased activity in right posterior superior temporal sulcus, right temporal pole, and superior temporal sulcus. Medial prefrontal cortex seemed to be involved in thinking about agents that were visible in the environment. |
| (Schweizer et al., 2013)  Canada | fMRI | | Quantitative descriptive study: case series(4)  Low risk of bias | Actively driving adults  9 M 7 F  ($\bar{X}$: 25.8y) | | Driving a car in a simulator | d4758 | 3 | To identify the neural underpinnings of human driving behavior by visualizing the areas of the brain involved in driving under different levels of demands | Regular driving: right turn; minimal significant activation, including somatosensory association (postcentral gyrus), parietal (precuneus), visual cortices (lingual gyrus).  Left turn; more activation in premotor cortex, somatosensory area, visual and parietal cortices, cerebellum. Left turn + traffic: larger significant activation in multiple bilateral regions in midposterior brain, including motor and premotor areas, visual parietal, somatosensory regions and cerebellum. Distracted driving: shift from posterior, visual and spatial areas to the prefrontal cortex. |
| (Wan et al., 2011)  Japan | fMRI | | Quantitative descriptive study: case series(4)  Low risk of bias | Right-handed professional Shogi players  11 M  ($\bar{X}$: 30y)  Low rank amateur players  9 M  ($\bar{X}$: 31.7y) | | Playing Shogi game | d9200 | 3 | To explore neural circuits that are specifically activated during the perception of- shogi patterns | Two activations for professionals: precuneus of parietal lobe during perception of board patterns, caudate nucleus of basal ganglia during quick generation of best move. |
| (Gatti et al., 2017)  Italy | fMRI | | Non- randomized trial (3B)  Low risk of bias | Right-handed adults selected on their daily activities in terms of hobbies and amusements  14 M 15 F  ($\bar{X}$: 23.4y) | | Executing and observing familiar and meaningless task, complex motor task and familiar meaningful task | d2101 | 2 | To define the presence and extent of activation of the motor system and the mirror neuron systems during motor tasks | Mirror neuron system activation was higher during finalistic motor task than simple motor task or complex motor task, inferior frontal gyrus recruitment was more significant during simple motor task than complex motor tasks in the execution and observation conditions.  Compared to simple motor and finalistic motor task, complex task resulted in increased recruitment of brain regions involved in complex motor task performance.  Watching meaningful actions resulted in increased connectivity with medio-prefrontal cortex. |
| (Walter et al., 2001)  Germany | fMRI | | Quantitative descriptive study: case series(4)  Low risk of bias | Right-handed adults who has a car driver license  6 M 6 F  (25-35y)  ($\bar{X}$: 29.9y) | | Driving actively and passively a car in simulator in virtual Hamburg | d4758 | 4 | To study brain regions correlated with car driving and to demonstrate that simulated car driving can be studied within a fMRI environments | Simulated driving requires the coordinated activity of occipito-parietal and motor brain areas. Motion sensitive areas are less active when comparing stimulated driving with passive driving. Activity associated with driving in sensorimotor cortex and cerebellum |
| (Karmonik et al., 2016) USA | fMRI | | Non randomized trial (3B)  Low risk of bias | Healthy adults with varying relationship to music  4 M 8 F  (18-82y) | | Listen to a self-selected musical piece that evokes a strong emotional response, two unfamiliar music pieces, and three spoken language pieces | d115 | 4 | To have a better understanding of changes in functional connectivity and information flow in order to optimize this approach through music therapy | The BOLD activation maps for all auditory pieces showed strong activation along the bilateral superior temporal gyrus and the bilateral inferior frontal gyrus. The self-selected emotional music showed the most pronounced activation (highest values in the BOLD activation maps relative to the silence periods) in regions of the midbrain (periaqueductal gray) as well as in the bilateral thalamus, bilateral caudate, bilateral lentiform nucleus, the bilateral parahippocampal gyrus, the substantia nigra, the anterior cingulate and posterior cingulate. Other areas included the bilateral medial frontal gyrus (including the supplemental motor area), the bilateral middle frontal gyrus, and regions in the cerebellum |
| (Bengtsson & Ullén, 2006)  Sweden | fMRI | | Quantitative descriptive study: non-comparative study, case series(4)  Low risk of bias | Right-handed healthy adults, professional pianists  11 M  (23-41y)  $(\bar{X}$: 33y) | | Participants read score without playing, while listening to four isochronous metronome beats providing tempo. Secondly, the participants start playing piano while continuing to read the musical score. | d9202 | 3 | To dissociate between brain areas processing the melodic and the rhythmic aspects during piano playing from musical scores | The medial occipital lobe, the superior temporal lobe, the rostral cingulate cortex, the putamen and the cerebellum process the melodic information, whereas the lateral occipital and the inferior temporal cortex, the left supramarginal gyrus, the left inferior and ventral frontal gyri, the caudate nucleus, and the cerebellum process the rhythmic information. |
| (Klasen, Weber, Kircher, Mathiak, & Mathiak, 2012)  Germany | fMRI | | Quantitative descriptive study; non-comparative study, case series(4)  Low risk of bias | Right-handed adults with gaming experiences  13 M  18-26 y | | Playing video game: ‘Tactival Ops: Assault on Terror’ | d9200 | 4 | To study brain correlates of content factors contributing to the experience of flow in a video game. | High vs low presence experience elicited patterns similar to those in the event-related conjunction encompassing somatosensory and premotor areas, superior parietal cortex, thalamus and cerebellum. Furthermore, inferior parietal lobe down regulations were observed during the phases of high presence comparable to the focus condition in the study. Flow demonstrated in midbrain reward structures, sensorimotor, cognitive and emotional brain circuits. |
| (Mathiak et al., 2011)  Germany | fMRI | | Quantitative descriptive study: non-comparative study, case series(4)  Low risk of bias | Right-handed adults with game experiences  13 M  (18-26y)  ($\bar{X}$: 22.7y) | | First-person shooter game (‘Tactical OPS: Assault on terror’) | d9200 | 5 | To address neuronal correlates of the rewarding aspect violence in a video game | Failure and success events evoked increased activity in visual cortex but only failure decreased activity in orbitofrontal cortex and caudate nucleus. Success events evoked increased activity in the cerebellum and decreased activity in the rostral anterior cingulate cortex. |
| (Pereira et al., 2011)  Portugal | fMRI | | Quantitative descriptive study: non-comparative study, case series(4)  Low risk of bias | Right-handed adults (music lovers)  9 M 5 F  (24-40y)  ($\bar{X}$: 32y) | | Listening to pop/rock music (familiar and liked, familiar and disliked, unfamiliar and liked, unfamiliar and disliked) | d115 | 3 | To clarify the role of familiarity in the brain correlates of music appreciation by controlling for both familiarity and musical preferences  To investigate how musical preferences and familiarity modulate the activity of brain regions recruited during music listening and appreciation | Broad emotion-related limbic and paralimbic regions as well as the reward circuitry were significantly more active for familiar relative to unfamiliar music. Smaller regions in the cingulate cortex and frontal lobe, including the motor cortex and Broca’s area, were found to be more active in response to liked music when compared to disliked one. |
| (Pinho, de Manzano, Fransson, Eriksson, & Ullén, 2014)  Sweden | fMRI | | Quantitative descriptive study: non-comparative study, case series(4)  Low risk of bias | Right-handed pianists  24 M 15 F  (19-67y)  ($\bar{X}$: 32.4y) | | Playing piano (keyboard), improvisation | d9202 | 5 | To measure brain activity during improvisation professional pianists with varying backgrounds in classical and jazz piano playing. | Total hours of improvisation experience was negatively associated with activity in frontoparietal executive cortical areas. In contrast, improvisation training was positively associated with functional connectivity of the bilateral dorsolateral prefrontal cortices, dorsal premotor cortices, and presupplementary areas. The effects were significant when controlling for hours of classical piano practice and age. |
|  |  | |  |  | |  |  |  |  |  |
| (Zhou, Wu, Zheng, Xiao, Zheng, 2022)  China | fMRI | | Quantitative non-randomized: non-randomized controlled trial  Low risk of bias | Right-handed students comprising music majors with >5 years of voice or instrument training  7 M 15 F  ($\bar{X}$: 23y)  Controls: non-musician students  7 M 15 F  ($\bar{X}$: 20.3y) | | Music excerpts of musical performance under audio only and audio-visual modality conditions. | d110-d115 | 5 | To explore the neural mechanism for the audio-visual integration of musical emotions | Regions with significant activation differences between audio only and audio visual; inferior parietal lobule, medial frontal gyrus, superior temporal gyrus, middle temporal gyrus, precuneus, posterior cingulate, lingual gyrus, parahippocampal gyrus. |
| (Talami, Vaudano, Meletti, 2020)  Italy | fMRI | | Quantitative descriptive study: case series(4)  Low risk of bias | Right-handed healthy volunteers  20 M 15 F  ($\bar{X}$: 18.4y) | | Viewing cartoons, movies or standup comedy that the participant selected as funny | d110-d115 | 5 | To characterize the neural correlates of emotional laughter | Laughter-related blood oxygen level-dependent (BOLD) increases involved both the motor (motor cortex, supplementary motor area, frontal operculum) and the emotional/limbic (anterior cingulate cortex, amygdala, n. accumbens, hippocampus) systems, as well  as modulatory circuitries encompassing the basal ganglia, thalamus, and cerebellum. developmental changes were identified in laughter processing, consisting in a greater  engagement of the reward circuitry in younger subjects; conversely, the default mode network appears more activated in older participants. |
| (Shane, Drover, Clingingsmith, Cerf, 2020)  USA | fMRI | | Quantitative descriptive study: case series(4)  Moderate risk of bias | Right-handed informal investors  10 M 5 F  ($\bar{X}$:29.6y) | | Viewing entrepreneur pitch video | d110-d115 | 5 | To explore how variation in entrepreneurs’ displayed passion affect informal investor interest in start-up ventures by examing neuronal responses to entrepreneurs’ | Notable functions that are activated by the high-passion pitch were the visual, auditory, semantic processing, emotion, and memory sites an well as cognitive processing the content. |
| (Jensen et al., 2020)  Sweden | fMRI | | Quantitative descriptive study: case series(4)  Low risk of bias | Physicians  8 M 10 F  (25-37y) | | Performing transcutaneous electrical nerve simulation treatment of pain to the arm in one of two patients. | d1608 | 4 | To determine the neural correlates of successful doctor-patient interactions | Brain regions where physicians displayed increased treatment-related activity when patients’ CARE ratings were high included medial orbitofrontal cortex (mOFC)/subgenual anterior cingulate cortex (sgACC) as well as the rostral anterior cingulate cortex (rACC)/ventromedial prefrontal cortex (vmPFC). |
| (Lee & Reeve, 2020)  Korea | fMRI | | Quantitative descriptive study: case series(4)  Low risk of bias | Right-handed undergraduates  9 M 9 F  ($\bar{X}$: 23y) | | Remembering intrinsic motivation or non-intrinsic motivation memories | d163 | 5 | To understand the underlying neural mechanism of intrinsic motivation memories | Results showed that both the ventromedial prefrontal cortex (VMPFC) and anterior cingulate cortex (ACC) were more  activated during the recall of intrinsically-motivating memories rather than during the recall of non-intrinsically-motivating  memories. Greater negative functional interactions between the VMPFC and ACC were also observed in the intrinsically motivating situations. These findings suggest that the two complementary neural processes are employed to reconstruct  intrinsically-motivating experiences: pleasure (reward related to VMPFC activity) and personal meaning (self-endorsement  related to ACC activity). |
| (Chen et al., 2017)  China | PET, fMRI | | Randomized controlled trial (1B)  Low risk of bias | Students who found Gangnam style pleasant  15 M  ($\bar{X}$: 24.1y) | | Listening to Gangnam style and light music | d115 | 4 | To use fMRI and PET for mapping neural changes under the most popular music in healthy volunteers | Increased fMRI BOLD signals in bilateral superior temporal cortices, left cerebellum, left putamen, right thalamus cortex. Monoamine receptor availability was increased in left superior temporal gyrus and left putamen, decreased in bilateral superior occipital cortices under Gangnam style compared with light music. Positive correlation between C-N-methylspiperone (C-NMSP) binding and BOLD signals in left temporal cortex. Increased C-NMSP binding in the left putamen was positive correlated with mood arousal level under Gangnam style condition. |
|  | |  | | | Note: if population is not specified, persons are healthy. M: males F: females $\bar{X}$: mean age Ex: experiment y: years Level of evidence was based on the OCEBM Levels of Evidence Working Group (2009). The Oxford Levels of Evidence. Oxford Centre for Evidence-Based Medicine. http://www.cebm.net/index.aspx?o=5653 | | | | | |

*Supplemental Table S1. PubMed search strategy*

| Research question: What is known in brain registration studies (brain functioning as measured by EEG, fNIRS, fMRI, PET) about meaningful activities in adults without neurologic pathologies?’  *Supplemental material: Table 1. PubMed search strategy* | | |
| --- | --- | --- |
| **NAME OF DATABASE (interface): PubMed** | | |
| **Concept** | **Line number** | **Search strategy ^^[[1]](#endnote-1)^^** |
| Concept 1: chronic disease | 1 | ((“Chronic disease”[Mesh] OR “chronic diseases”[TIAB] OR “Noncommunicable diseases”[Mesh] OR “noncommunicable disease”[TIAB] OR “non-communicable diseases”[TIAB] OR “multiple chronic conditions”[TIAB] OR “chronic illnesses”[TIAB] OR “chronic illness”[TIAB] OR “multiple chronic disease”[TIAB] OR “multiple chronic diseases”[TIAB] OR “non-communicable chronic diseases”[TIAB] OR “non communicable chronic disease”[TIAB] OR “multiple chronic health conditions”[TIAB] OR “multiple chronic medical conditions”[TIAB] OR “chronic obstructive pulmonary disease”[TIAB] OR “hereditary autoinflammatory diseases”[TIAB] OR “hereditary autoinflammatory disease”[TIAB] OR “chronic fatigue syndrome”[TIAB] OR “chronic kidney failure”[TIAB] OR “leukemia”[TIAB] OR “cancer”[TIAB] OR “Diabetes”[TIAB] OR “arthritis”[TIAB] OR “Tuberculosis”[TIAB] OR “HIV”[TIAB] OR “long-term illness”[TIAB] OR “long term illnesses”[TIAB] OR “chronic health conditions”[TIAB] OR “chronic health condition”[TIAB] OR “chronic ailments”[TIAB] OR “chronically ill”[TIAB]) NOT (“stroke”[TIAB] OR “cerebrovascular accident”[TIAB] OR “cerebrovascular disease”[TIAB] OR “acute stroke”[TIAB] OR “post-stroke”[TIAB] OR “ischemic attack”[TIAB] OR “CVA”[TIAB] OR “mental disease”[TIAB] OR “mental disorder”[TIAB] OR “schizophrenia”[TIAB] OR “neurosis”[TIAB] OR “dementia”[TIAB] OR “Alzheimer”[TIAB])) |
| Concept 2: healthy persons | 2 | (“healthy people”[TIAB] OR “healthy volunteers”[TIAB] OR “healthy individuals”[TIAB] OR “healthy persons”[TIAB] OR “active people”[TIAB] OR “normal people”[TIAB] OR “normal human”[TIAB] OR “healthy human”[TIAB] OR “normal conditions”[TIAB] OR “normal condition”[TIAB] OR “good health”[TIAB]) |
| Concept 3: brain imaging | 3 | (“Neuroimaging”[Majr] OR “brain imaging”[TIAB] OR “neuroimaging”[TIAB] OR “functional neuroimaging”[TIAB] OR “Three-dimensional imaging”[TIAB] OR “fMRI”[TIAB] OR “functional magnetic resonance imaging”[TIAB] OR (“magnetic resonance”[TIAB] AND (“image”[TIAB] OR “images”[TIAB] OR “imaging”[TIAB])) OR “fNIRS”[TIAB] OR “functional near infrared spectroscopy”[TIAB] OR “EEG”[TIAB] OR “electroencephalogram”[TIAB] OR “near-infrared imaging”[TIAB] OR “functional MRI”[TIAB] OR “MRI scan”[TIAB] OR “brain waves”[TIAB] OR “neuroimaging activity”[TIAB]) |
| Concept 4: meaningful activities | 4 | (“activities of daily living”[Majr] OR “activities of daily life”[TIAB] OR “activity of daily life”[TIAB] OR “ADL”[TIAB] OR “well-being”[TIAB] OR “wellbeing”[TIAB] OR “engagement”[TIAB] OR “meaningful actions”[TIAB] OR “meaningful action”[TIAB] OR “meaningful occupations”[TIAB] OR “meaningful occupation”[TIAB] OR “occupation”[TIAB] OR “occupations”[TIAB] OR “hobby”[TIAB] OR “hobbies”[TIAB] OR “spirituality”[TIAB] OR “meaningful life”[TIAB] OR “meaningful living”[TIAB] OR “leisure activities”[TIAB] OR “leisure activity”[TIAB] OR “activity of living”[TIAB] OR “activities of living”[TIAB] OR “daily life activities”[TIAB] OR “daily life activity”[TIAB] OR “social participation”[Majr] OR “tailored activity”[TIAB] OR ”tailored activities”[TIAB] OR “daily activity”[TIAB] OR “daily activities”[TIAB] OR “self-care”[TIAB] OR “preferred activity”[TIAB] OR “preferred activities”[TIAB] OR “preferred occupations”[TIAB] OR “preferred occupation”[TIAB] OR “pleasure”[TIAB] OR “individualized tailored activity”[TIAB] OR “individualised tailored activity”[TIAB] OR “individualised tailored activities”[TIAB] OR “individualized tailored activities”[TIAB] OR “preferred occupation”[TIAB] OR “preferred activity”[TIAB] OR “preferred activities”[TIAB] OR “activity satisfaction”[TIAB] OR “daily living activity”[TIAB] OR “daily living activities”[TIAB] OR “iADL”[TIAB] OR “instrumental activities of daily life”[TIAB] OR “instrumental activities of daily living”[TIAB] OR “daily function”[TIAB] OR “daily functioning”[TIAB] OR “daily functions”[TIAB]) |
| Filter / search block: |  | Year of publication: 2000-2022 |
|  | 4/09/22 | (1 OR 2 ) AND 3 AND 4 |

*Supplementary Table S2. linking ruler ICF*

| **Author** | **Study activity** | **Name ICF code** | **Component** | **1st level chapter** | **2nd level** | **3rd level** |
| --- | --- | --- | --- | --- | --- | --- |
| (Blood & Zatorre, 2001) Canada | Listening to self-selected pleasant music | Listening | D | 1 | 15 |  |
| (Small, Zatorre, Dagher, Evans, & Jones-Gotman, 2001) Canada | Eating chocolate | Eating | D | 5 | 50 |  |
| (Matsunaga et al., 2014) Japan | Watching an emotionally neutral film (control) or film featuring people the participants considered attractive | Watching | D | 1 | 10 |  |
| (Perreau-Linck et al., 2007) Canada | Recalling autobiographical memories (happiness, sadness, neutral) | Thinking | D | 1 | 63 |  |
| Jin et al., 2020 | Melodic recall and execution of tapping | Thinking | D  D | 1  2 | 6  2 | 3  0 |
| Chabin et al., 2020 | Listen to their favorite pleasurable chill-inducing musical excerpts | Listening | D | 1 | 1 | 5 |
| Ara & Marco-Pallarés (2020) | Listen to commercially available songs of several music genres | Listening | D | 1 | 1 | 5 |
| Marion et al., 2021 | Listen to melodies while reading the musical score  Imagine the melody in sync with the tactile metronome | Listening | D  D | 1  1 | 1  6 | 5  3 |
| (Moghimi, Kushki, Power, Guerguerian, & Chau, 2012)  Canada | Listening to music, with some self-selected musical pieces | Listening | D | 1 | 15 |  |
| (Balardin et al., 2017) Brazil | Ex1: table tennis  Ex2: playing piano  Ex3: interaction violin  Ex4: daily work activities | Sports  Arts and culture  Idem  Completing the daily routine | D  D  D | 9  9  2 | 20  20  30 | 1  2  2 |
| (Verdiere, Roy, & Dehais, 2018) France | Manual and automated landing in flight simulator | Driving motorized vehicles | d | 4 | 75 | 1 |
| Chang et al., 2021 | Create a video using IMovie | Applying knowledge | D | 1 | 9 | 7 |
| Pan et al., 2021 | Mastering two Chinese ancient poems (cooperatively or independently) | Thinking | D  D | 1 | 6 | 3  6 |
| (Boissoneault, Sevel, Robinson, & Staud, 2018) USA | Recall a fatigue and happy experience | Thinking | D | 1 | 63 |  |
| (Spiers & Maguire, 2006) UK | ‘The Getaway’ game, driving a virtual cab with customers in London | Driving, other specified | D | 4 | 75 | 8 |
| (T. A. Schweizer et al., 2013) Canada | Driving a car in a simulator | Driving, other specified | D | 4 | 75 | 8 |
| (Wan et al., 2011) Japan | Playing Shogi game | Play | D | 9 | 20 | 0 |
| (Gatti et al., 2017) Italy | Executing and observing familiar and meaningless task, complex motor task and familiar meaningful task | Undertaking a complex task | D | 2 | 10 | 1 |
| (Walter et al., 2001) Germany | Driving actively and passively a car in simulator in virtual Hamburg | Driving, other specified | D | 4 | 75 | 8 |
| (Karmonik et al., 2016) USA | Listen to a self-selected musical piece that evokes a strong emotional response, two unfamiliar music pieces, and three spoken language pieces | Listening | D | 1 | 15 |  |
| (Bengtsson & Ullén, 2006) Sweden | Participants read score without playing, while listening to four isochronous metronome beats providing tempo. Secondly, the participants start playing piano while continuing to read the musical score. | Art and culture | d | 9 | 20 | 2 |
| (Klasen, Weber, Kircher, Mathiak, & Mathiak, 2012) Germany | Playing video game: ‘Tactival Ops: Assault on Terror’ | Play | D | 9 | 20 | 0 |
| (Mathiak et al., 2011) Germany | First-person shooter game (‘Tactical OPS: Assault on terror’) | Play | D | 9 | 20 | 0 |
| (Pereira et al., 2011) Portugal | Listening to pop/rock music (familiar and liked, familiar and disliked, unfamiliar and liked, unfamiliar and disliked) | Listening | D | 1 | 15 |  |
| (Pinho, de Manzano, Fransson, Eriksson, & Ullén, 2014) Sweden | Playing piano (keyboard), improvisation | Art and culture | d | 9 | 20 | 2 |
| Zhou et al., 2022 | Listen and watching music performance | Listening  watching | D | 1 | 1 | 0  5 |
| Talami et al., 2020 | Watching cartoons, movies or stand up comedy | Watching  listening | D | 1 | 1 | 0  5 |
| Shane et al., 2020 | Viewing entrepreneur pitch video | Watching  listening | D | 1 | 1 | 0  5 |
| Jensen et al., 2020 | Performing transcutaneous electrical nerve simulation treatment of pain to the arm in one of two patients. | Focusing attention, other specified | D | 1 | 6 | 08 |
| Lee & Reeves, 2020 | To remember intrinsic motivation or non-intrinsic motivation memories | thinking | D | 1 | 6 | 3 |
| (Chen et al., 2017) China | Listening to Gangnam style and light music | Listening | D | 1 | 15 |  |

*Supplementary Table S3 MMAT*

| **Authors** | Questions for each design |
| --- | --- |
| (Balardin et al., 2017)  **(Case-series)** | S1. Are there clear research questions? yes  S2. Do the collected data allow to address the research questions? yes  4.1. Is the sampling strategy relevant to address the research question? Non-probability sampling, NO  4.2. Is the sample representative of the target population? NO  4.3. Are the measurements appropriate? Yes  4.4. Is the risk of nonresponse bias low? yes  4.5. Is the statistical analysis appropriate to answer the research question? Yes  🡪 score 3 |
| (Verdiere, Roy, & Dehais, 2018) **(case series)** | S1. Are there clear research questions? yes  S2. Do the collected data allow to address the research questions? yes  4.1. Is the sampling strategy relevant to address the research question? yes  4.2. Is the sample representative of the target population? NO ( young group men)  4.3. Are the measurements appropriate? yes  4.4. Is the risk of nonresponse bias low? yes  4.5. Is the statistical analysis appropriate to answer the research question? Yes  Score: 4 |
| (Boissoneault, Sevel, Robinson, & Staud, 2018) **(quantitative descriptive : case series)** | S1. Are there clear research questions?  S2. Do the collected data allow to address the research questions?  4.1. Is the sampling strategy relevant to address the research question? yes  4.2. Is the sample representative of the target population? no  4.3. Are the measurements appropriate? yes  4.4. Is the risk of nonresponse bias low? yes  4.5. Is the statistical analysis appropriate to answer the research question? Yes  🡪 4 |
| (Spiers & Maguire, 2006) **(case series)** | S1. Are there clear research questions? yes  S2. Do the collected data allow to address the research questions? yes  4.1. Is the sampling strategy relevant to address the research question? yes  4.2. Is the sample representative of the target population? No (only men)  4.3. Are the measurements appropriate? yes  4.4. Is the risk of nonresponse bias low? yes  4.5. Is the statistical analysis appropriate to answer the research question? Yes  Score: 4 |
| (Schweizer et al., 2013) **(case series)** | S1. Are there clear research questions? yes  S2. Do the collected data allow to address the research questions? Yes  4.1. Is the sampling strategy relevant to address the research question? no  4.2. Is the sample representative of the target population? no  4.3. Are the measurements appropriate? yes  4.4. Is the risk of nonresponse bias low? yes  4.5. Is the statistical analysis appropriate to answer the research question? Yes  🡪 score: 3 |
| (Wan et al., 2011)  **(case report)** | S1. Are there clear research questions? yes  S2. Do the collected data allow to address the research questions? Yes  4.1. Is the sampling strategy relevant to address the research question? Can’t tell  4.2. Is the sample representative of the target population? can’t tell, no information on age  4.3. Are the measurements appropriate? yes  4.4. Is the risk of nonresponse bias low? yes  4.5. Is the statistical analysis appropriate to answer the research question? yes  🡪 score: 3 |
| (Gatti et al., 2017)  **(randomized trial)** | S1. Are there clear research questions? yes  S2. Do the collected data allow to address the research questions? yes  .1. Is randomization appropriately performed? No  2.2. Are the groups comparable at baseline? yes  2.3. Are there complete outcome data? yes  2.4. Are outcome assessors blinded to the intervention provided? no  2.5 Did the participants adhere to the assigned intervention? No, some participants were in more than one group.  🡪 score: 2 |
| (Walter et al., 2001)  **(case series)** | S1. Are there clear research questions? yes  S2. Do the collected data allow to address the research questions? yes  4.1. Is the sampling strategy relevant to address the research question? yes  4.2. Is the sample representative of the target population? no  4.3. Are the measurements appropriate? yes  4.4. Is the risk of nonresponse bias low? yes  4.5. Is the statistical analysis appropriate to answer the research question? Yes  🡪 score 4 |
| (Karmonik et al., 2016) **(quantitative non-randomized)** | S1. Are there clear research questions? yes  S2. Do the collected data allow to address the research questions? yes  Are the participants representative of the target population? yes  Are measurements appropriate regarding both the outcome and intervention (or exposure)? yes  Are there complete outcome data? yes  3.4. Are the confounders accounted for in the design and analysis? yes  3.5 During the study period, is the intervention administered (or exposure occurred) as intended? No  🡪 score: 4 |
| (Blood & Zatorre, 2001) **(case series)** | Quantitative descriptive   \| S1 \| yes \| \| --- \| --- \| \| S2 \| yes \| \| 1 \| yes \| \| 2 \| yes \| \| 3 \| yes \| \| 4 \| yes \| \| 5 \| yes \| |
| (Small et al., 2001)**(Case series)** | Quantitative descriptive   \| S1 \| yes \| \| --- \| --- \| \| S2 \| yes \| \| 1 \| yes \| \| 2 \| yes \| \| 3 \| yes \| \| 4 \| yes \| \| 5 \| yes \| |
| (Matsunaga et al., 2014)**( non-randomized controlled trial)** | Quantitative non-randomised   \| S1 \| yes \| \| --- \| --- \| \| S2 \| yes \| \| 1 \| yes \| \| 2 \| yes \| \| 3 \| ? \| \| 4 \| yes \| \| 5 \| yes \| |
| (Perreau-Linck et al.,2007) **(case series)** | Quantitative descriptive   \| S1 \| yes \| \| --- \| --- \| \| S2 \| yes \| \| 1 \| yes \| \| 2 \| yes \| \| 3 \| yes \| \| 4 \| yes \| \| 5 \| yes \| |
| (Moghimi, Kushki, Power, Guerguerian, & Chau, 2012)**(case series)** | Quantitative descriptive   \| S1 \| yes \| \| --- \| --- \| \| S2 \| yes \| \| 1 \| yes \| \| 2 \| yes \| \| 3 \| yes \| \| 4 \| yes \| \| 5 \| yes \| |
| (Mathiak et al., 2011)(**case series)** | 4.1 YES  4.2 YES  4.3 YES  4.4 YES  4.5 YES |
| ( Pereira et al., 2011) **(case series)** | 4.1 NO  4.2 YES  4.3 YES  4.4 NO  4.5 YES |
| (Chen et al., 2017) **(RCT)** | 2.1 Can’t tell  2.2 YES  2.3 YES  2.4 YES  2.5 YES |
| (Schweizer et al, 2013)**(case series)** | 4.1 YES  4.2 YES  4.3 YES  4.4 YES  4.5 YES |
| (Pinho et al., 2014)**(case-series)** | 4.1 YES  4.2 YES  4.3 YES  4.4 YES  4.5 YES |
| (Klasen et al., 2012)**(Case series)** | 4.1 YES  4.2 NO  4.3 YES  4.4 YES  4.5 YES |
| (Bengtsson and Ullén, 2006)**(Case series)** | 4.1 Can’t tell  4.2 NO: no women  4.3 YES  4.4 YES  4.5 YES |
| (Chabin et al., 2020)  **(Quantitative descriptive)** | S1. Are there clear research questions? yes  S2. Do the collected data allow to address the research questions? yes  4.1. Is the sampling strategy relevant to address the research question? yes  4.2. Is the sample representative of the target population? yes  4.3. Are the measurements appropriate? yes  4.4. Is the risk of nonresponse bias low? yes  4.5. Is the statistical analysis appropriate to answer the research question? yes  Score:5 |
| (Jin et al., 2021)  **(Quantitative nonrandomized)** | S1. Are there clear research questions? YES  S2. Do the collected data allow to address the research questions? YES  3.1. Are the participants representative of the target population? YES  3.2. Are measurements appropriate regarding both the outcome and intervention (or exposure)? YES  3.3. Are there complete outcome data? YES  3.4. Are the confounders accounted for in the design and analysis? yes  3.5. During the study period, is the intervention administered (or exposure occurred) as intended? yes  5 |
| (Ara et al., 2020)  **(Quantitative descriptive)** | S1. Are there clear research questions? YES  S2. Do the collected data allow to address the research questions? YES  4.1. Is the sampling strategy relevant to address the research question? YES  4.2. Is the sample representative of the target population? YES  4.3. Are the measurements appropriate? YES  4.4. Is the risk of nonresponse bias low? YES  4.5. Is the statistical analysis appropriate to answer the research question? YES  Score:5 |
| (Marion et al., 2021)  **(Quantitative descriptive)** | S1. Are there clear research questions? yes  S2. Do the collected data allow to address the research questions? no  4.1. Is the sampling strategy relevant to address the research question? yes  4.2. Is the sample representative of the target population? yes  4.3. Are the measurements appropriate? yes  4.4. Is the risk of nonresponse bias low? yes  4.5. Is the statistical analysis appropriate to answer the research question? no  Score:4 |
| (Chang et al., 2021)  **(Quantitative descriptive)** | S1. Are there clear research questions? YES  S2. Do the collected data allow to address the research questions? YES  4.1. Is the sampling strategy relevant to address the research question? YES  4.2. Is the sample representative of the target population? YES  4.3. Are the measurements appropriate? YES  4.4. Is the risk of nonresponse bias low? YES  4.5. Is the statistical analysis appropriate to answer the research question? NO  Score:4 |
| (Pan, et al., 2021)  **(Quantitative descriptive)** | S1. Are there clear research questions? yes  S2. Do the collected data allow to address the research questions? yes  4.1. Is the sampling strategy relevant to address the research question? yes  4.2. Is the sample representative of the target population? no  4.3. Are the measurements appropriate? yes  4.4. Is the risk of nonresponse bias low? yes  4.5. Is the statistical analysis appropriate to answer the research question? yes  Score:4 |
| (Zhou et al., 2022)  **(Quantitative nonrandomized)** | S1. Are there clear research questions? no  S2. Do the collected data allow to address the research questions? yes  3.1. Are the participants representative of the target population? yes  3.2. Are measurements appropriate regarding both the outcome and intervention (or exposure)? yes  3.3. Are there complete outcome data? yes  3.4. Are the confounders accounted for in the design and analysis? yes  3.5. During the study period, is the intervention administered (or exposure occurred) as intended? yes |
| (Talami et al., 2020)  **(Quantitative descriptive)** | S1. Are there clear research questions? YES  S2. Do the collected data allow to address the research questions? YES  4.1. Is the sampling strategy relevant to address the research question? +  4.2. Is the sample representative of the target population? YES  4.3. Are the measurements appropriate? YES  4.4. Is the risk of nonresponse bias low? YES  4.5. Is the statistical analysis appropriate to answer the research question? YES  Score:5 |
| (Shane et al., 2020)  **(Quantitative descriptive)** | S1. Are there clear research questions? YES  S2. Do the collected data allow to address the research questions? YES  4.1. Is the sampling strategy relevant to address the research question? YES  4.2. Is the sample representative of the target population? YES  4.3. Are the measurements appropriate? YES  4.4. Is the risk of nonresponse bias low? YES  4.5. Is the statistical analysis appropriate to answer the research question? YES  Score:5 |
| (Jensen et al., 2020)  **(Quantitative descriptive)** | S1. Are there clear research questions? NO  S2. Do the collected data allow to address the research questions? YES  4.1. Is the sampling strategy relevant to address the research question? YES  4.2. Is the sample representative of the target population? YES  4.3. Are the measurements appropriate? NO  4.4. Is the risk of nonresponse bias low? YES  4.5. Is the statistical analysis appropriate to answer the research question? YES  Score:4 |
| (Lee & Reeve, 2020)  **(Quantitative descriptive)** | S1. Are there clear research questions? YES  S2. Do the collected data allow to address the research questions? YES  4.1. Is the sampling strategy relevant to address the research question? YES  4.2. Is the sample representative of the target population? YES  4.3. Are the measurements appropriate? YES  4.4. Is the risk of nonresponse bias low? YES  4.5. Is the statistical analysis appropriate to answer the research question? YES  Score:5 |

*Supplementary* Table S4. Critical Appraisals Tool (Joanna Briggs Institute)

Case series

|  | (Blood et al., 2001) | (Small et al.,2001) | (Perreau-Linck et al., 2007) | (Olszewska-Guizzo et al., 2018) | (Moghimi et al., 2012) | (Balardin et al., 2017) |
| --- | --- | --- | --- | --- | --- | --- |
| Were there clear criteria for inclusion in the case series? | / | + | / | / | - | / |
| Was the condition measured in a standard, reliable way for all participants included in the case series? | + | + | + | + | + | - |
| Were valid methods used for identification of the condition for all participants included in the case series? | + | + | + | + | + | + |
| Did the case series have consecutive inclusion of participants? | _ | + | + | + | - | + |
| Did the case series have complete inclusion of participants? | + | + | - | + | + | + |
| Was there clear reporting of the demographics of the participants in the study? | + | _ | + | + | + | + |
| Was there clear reporting of clinical information of the participants? | / | + | + | + | + | + |
| . Were the outcomes or follow up results of cases clearly reported? | + | + | - | + | + | + |
| Was there clear reporting of the presenting site(s)/clinic(s) demographic information? | / | _ | + | + | + | - |
| Was statistical analysis appropriate? | + | + | + | + | + | - |
| Risk of bias  Low/moderate/severe  Scoring for overall risk-of-bias assessment is as follows: 0–3 minuses, low risk of bias (L); 4–6 minuses, moderate risk of bias (M); 7–9 minuses, high risk of bias (H). | low | Low | Low | Low | low | Low |

|  | (Verdiere et al., 2018) | (Boissoneault et al., 2018) | Spiers & Maguire, 2006) | (Schweizer et al., 2013) | (Wan et al., 2011) | (Walter et al., 2001) | (Bengtsson & Ullén, 2006) | (Klasen et al., 2012) |
| --- | --- | --- | --- | --- | --- | --- | --- | --- |
| Were there clear criteria for inclusion in the case series? | / | + | + | + | / | + | + | + |
| Was the condition measured in a standard, reliable way for all participants included in the case series? | + | + | + | + | + | + | + | - |
| Were valid methods used for identification of the condition for all participants included in the case series? | + | + | + | + | + | + | + | + |
| Did the case series have consecutive inclusion of participants? | - | + | + | + | - | + | + | - |
| Did the case series have complete inclusion of participants? | - | + | + | + | + | - | + | + |
| Was there clear reporting of the demographics of the participants in the study? | + | - | + | + | - | + | + | - |
| Was there clear reporting of clinical information of the participants? | + | / | + | + | - | + | + | / |
| Were the outcomes or follow up results of cases clearly reported? | + | + | + | + | + | + | + | + |
| Was there clear reporting of the presenting site(s)/clinic(s) demographic information? | + | + | + | + | + | + | + | + |
| Was statistical analysis appropriate? | + | + | + | + | + | + | + | + |
| Risk of bias | Low | Low | Low | Low | Low | Low | Low | Low |

|  | (Mathiak et al., 2011) | (Pereira et al., 2011) | (Pinho et al., 2014) |
| --- | --- | --- | --- |
| Were there clear criteria for inclusion in the case series? | / | / | + |
| Was the condition measured in a standard, reliable way for all participants included in the case series? | + | + | + |
| Were valid methods used for identification of the condition for all participants included in the case series? | + | + | + |
| Did the case series have consecutive inclusion of participants? | + | - | + |
| Did the case series have complete inclusion of participants? | - | + | + |
| Was there clear reporting of the demographics of the participants in the study? | + | - | + |
| Was there clear reporting of clinical information of the participants? | - | + | + |
| Were the outcomes or follow up results of cases clearly reported? | + | + | + |
| Was there clear reporting of the presenting site(s)/clinic(s) demographic information? | + | + | + |
| Was statistical analysis appropriate? | + | + | + |
| Conclusion risk of bias | low | Low | low |

| Case-controls | (Gatti et al., 2017) | (Karmonik et al., 2016) |
| --- | --- | --- |
| 1. Were the groups comparable other than the presence of disease in cases or the absence of disease in controls? | + | + |
| 1. Were cases and controls matched appropriately? | - | / |
| 1. Were the same criteria used for identification of cases and controls? | + | + |
| 1. Was exposure measured in a standard, valid and reliable way? | + | + |
| 1. Was exposure measured in the same way for cases and controls? | + | + |
| 1. Were confounding factors identified? | / | / |
| 1. Were strategies to deal with confounding factors stated? | / | / |
| 1. Were outcomes assessed in a standard, valid and reliable way for cases and controls? | + | + |
| 1. Was the exposure period of interest long enough to be meaningful? | + | + |
| 1. Was appropriate statistical analysis used? | + | + |
| conclusion | Low | Low |

| Case controls | Chen et al., 2017 |
| --- | --- |
| 1. Were the groups comparable other than the presence of disease in cases or the absence of disease in controls? | + |
| 1. Were cases and controls matched appropriately? | / |
| 1. Were the same criteria used for identification of cases and controls? | + |
| 1. Was exposure measured in a standard, valid and reliable way? | + |
| 1. Was exposure measured in the same way for cases and controls? | + |
| 1. Were confounding factors identified? | / |
| 1. Were strategies to deal with confounding factors stated? | / |
| 1. Were outcomes assessed in a standard, valid and reliable way for cases and controls? | + |
| 1. Was the exposure period of interest long enough to be meaningful? | + |
| 1. Was appropriate statistical analysis used? | + |
| - conclusion | low |

| Case series | Chabin et al., 2020 | Ara, Marco-Pallarés, 2020 | Marion, Di Liberto, Shamma, 2021 | Chang & Bruce, 2021 | Pan, Cheng, Hu, 2021) |
| --- | --- | --- | --- | --- | --- |
| Were there clear criteria for inclusion in the case series? | + | + | + | + | + |
| Was the condition measured in a standard, reliable way for all participants included in the case series? | + | + | / | + | / |
| Were valid methods used for identification of the condition for all participants included in the case series? | + | + | _ | + | + |
| Did the case series have consecutive inclusion of participants? | + | + | + | + | + |
| Did the case series have complete inclusion of participants? | + | + | _ | + | + |
| Was there clear reporting of the demographics of the participants in the study? | + | _ | _ | + | + |
| Was there clear reporting of clinical information of the participants? | + | / | / | / | / |
| Were the outcomes or follow up results of cases clearly reported? | + | + | + | _ | + |
| Was there clear reporting of the presenting site(s)/clinic(s) demographic information? | + | + | _ | + | + |
| Was statistical analysis appropriate? | + | + | + | _ | + |
| Conclusion risk of bias | low | LOW | MODERATE | LOW | low |

|  | | | Talami, Vaudan, Meletti, 2020 | | Shane et al., 2020 | Jensen et al., 2020 | | Lee & Reeve, 2020 |
| --- | --- | --- | --- | --- | --- | --- | --- | --- |
| Were there clear criteria for inclusion in the case series? | | | - | | + | _ | | + |
| Was the condition measured in a standard, reliable way for all participants included in the case series? | | | + | | _ | + | | / |
| Were valid methods used for identification of the condition for all participants included in the case series? | | | / | | _ | + | | / |
| Did the case series have consecutive inclusion of participants? | | | + | | + | + | | + |
| Did the case series have complete inclusion of participants? | | | + | | + | _ | | + |
| Was there clear reporting of the demographics of the participants in the study? | | | + | | ++ | _ | | _ |
| Was there clear reporting of clinical information of the participants? | | | + | | / | + | | / |
| Were the outcomes or follow up results of cases clearly reported? | | | + | | _ | _ | | + |
| Was there clear reporting of the presenting site(s)/clinic(s) demographic information? | | | + | | + | / | | / |
| Was statistical analysis appropriate? | | | + | | + | + | | + |
| Conclusion risk of bias | | | low | | MODERATE | LOW | | LOW |
| Case controls | Jin et al., 2020 | | Zhou et al., 2022 | | |  |  |  |
| 1. Were the groups comparable other than the presence of disease in cases or the absence of disease in controls? | + | | + | | |  |  |  |
| 1. Were cases and controls matched appropriately? | + | | _ | | |  |  |  |
| 1. Were the same criteria used for identification of cases and controls? | / | | + | | |  |  |  |
| 1. Was exposure measured in a standard, valid and reliable way? | + | | + | | |  |  |  |
| 1. Was exposure measured in the same way for cases and controls? | + | | + | | |  |  |  |
| 1. Were confounding factors identified? | + | | + | | |  |  |  |
| 1. Were strategies to deal with confounding factors stated? | - | | _ | | |  |  |  |
| 1. Were outcomes assessed in a standard, valid and reliable way for cases and controls? | + | | + | | |  |  |  |
| 1. Was the exposure period of interest long enough to be meaningful? | + | | + | | |  |  |  |
| 1. Was appropriate statistical analysis used? | + | | + | | |  |  |  |
| conclusion | Low risk | | LOW | | |  |  |  |

*Supplementary Table S5: References per theme that are stated in the result sections*

| **1. Brain registration technique** |  |
| --- | --- |
| See for references per brain registration Table 1 | |
| **2. Aim of the studies** | References |
| To find the neural network or the selected regions of interest in a specific activity. | Bengtsson & Ullén, 2006; Boissoneault et al., 2018; Gatti et al., 2017; Karmonik et al., 2016; Pinho et al., 2014; Schweizer et al., 2013; Spiers & Maguire, 2006; Verdiere et al., 2018; Walter et al., 2001; Wan et al., 2011; Jin et al., 2020; Ara & Marco-Pallarés, 2020; Marion, Di Liberto, Shamma, 2021; Chang, Seccia and Bruce, 2021; Pan, Cheng, Hu, 2021; Jensen et al., 2020 |
| To find the neural mechanisms underlying emotional responses related to the given activity. | Blood & Zatorre, 2001; Matsunaga et al., 2014; Moghimi et al., 2012; Perreau-Linck et al., 2007; Zhou et al., 2022; Talami et al., 2020; Shane et al., 2020 |
| To find the motivational region in the brain. | Klasen et al., 2012; Small et al., 2001 |
| To find in the brain what the preferences were of the participants in certain activities. | Pereira et al., 2011; |
| To evaluate the feasibility of the brain registration technique. | Balardin et al., 2017; Chen et al., 2017; Walter et al., 2001; Chabin et al., 2020 |
| To search the reward area in the brain. | Mathiak et al., 2011; |

| **3. Population** | References |
| --- | --- |
| Including gender equally | Blood & Zatorre, 2001; Moghimi et al., 2012; Walter et al., 2001; Lee, & Reeve, 2020 |
| Specified right-handed adults | Balardin et al., 2017; Bengtsson & Ullén, 2006; Gatti et al., 2017; Klasen et al., 2012; Mathiak et al., 2011; Matsunaga et al., 2014; Pereira et al., 2011; Pinho et al., 2014; Small et al., 2001; Walter et al., 2001; Wan et al., 2011; Jin et al., 2020; Chabin et al., 2020; Ara & Marco-Pallarés, 2020; Pan, Cheng, & Hu, 2021; Zhou, Wu, Zheng, Xiao, Zheng, 2022; Talami, Vaudano, Meletti, 2020; Shane, Drover, Clingingsmith, Cerf, 2020; Lee & Reeve, 2020 |
| Specified their participants as healthy students who conducted the activity | Blood & Zatorre, 2001; Pan, Cheng, Hu, 2021; Zhou et al., 2022; Lee & Reeve, 2020; Chen et al., 2017 ; Matsunaga et al., 2014 |
| **4. Type of activities** | References |
| Operating the main activity in a passive way | Blood & Zatorre, 2001; Boissoneault et al., 2018; Chen et al., 2017; Gatti et al., 2017; Karmonik et al., 2016; Matsunaga et al., 2014; Moghimi et al., 2012; Pereira et al., 2011; Perreau-Linck et al., 2007; Chabin et al., 2020 Ara & Marco-Pallarés, 2020; Marion et al., 2021; Pan et al., 2021; Zhou et al., 2022; Talami et al., 2020 ; Shane et al., 2020 ; Lee & Reeve, 2020 |
| Listening to music | Blood & Zatorre, 2001; Chen et al., 2017; Karmonik et al., 2016; Moghimi et al., 2012; Pereira et al., 2011; Chabin et al., 2020; Ara & Marco-Pallarés, 2020; Marion et al., 2021; Zhou et al., 2022 |
| Recalling a memory | Boissoneault et al., 2018; Perreau-Linck et al., 2007; Jin et al., 2020; Lee & Reeve, 2020 |
| Involving watching and reflecting | Gatti et al., 2017; Matsunaga et al., 2014; Pan et al., 2021 ; Talami et al., 2020 ; Shane et al., 2020 |
| Active way of performing a physical activity | Balardin et al., 2017; Bengtsson & Ullén, 2006; Gatti et al., 2017; Klasen et al., 2012; Mathiak et al., 2011; Pinho et al., 2014; Schweizer et al., 2013; Small et al., 2001; Spiers & Maguire, 2006; Verdiere et al., 2018; Walter et al., 2001; Wan et al., 2011; Chang et al., 2021; Jensen et al., 2020; Jin et al., 2020 |
| Studies performed in the lab | Bengtsson & Ullén, 2006; Blood & Zatorre, 2001; Boissoneault et al., 2018; Chen et al., 2017; Gatti et al., 2017; Karmonik et al., 2016; Klasen et al., 2012; Mathiak et al., 2011; Matsunaga et al., 2014; Moghimi et al., 2012; Pereira et al., 2011; Perreau-Linck et al., 2007; Pinho et al., 2014; Schweizer et al., 2013; Small et al., 2001; Spiers & Maguire, 2006; Verdiere et al., 2018; Walter et al., 2001; Wan et al., 2011; Jin et al., 2020; Chabin et al., 2020; Ara et al., 2020; Marion et al., 2021; Chang et al., 2021; Pan et al., 2021; Zhou et al., 2022; Talami et al., 20202; Shane et al., 2020; Jensen et al., 2020; Lee et al., 2020; |
| Activities performed in real circumstances | Balardin et al., 2017 |
| **ICF categories** | **References** |
| D1 *learning and applying knowledge* | Blood & Zatorre, 2001; Boissoneault et al., 2018;Chen et al., 2017; Karmonik et al., 2016; Matsunaga et al., 2014; Moghimi et al., 2012; Pereira et al., 2011; Perreau-Linck et al., 2007; Jin et al., 2020; Chabin et al., 2020; Ara et al., 2020; Marion et al., 2021; Chang et al., 2021; Pan et al., 2021; Zhou et al., 2022; Talami et al., 2020; Shane et al., 2020; Jensen et al., 2020; Lee et al., 2020 |
| D2 *general tasks and demands* | Balardin et al., 2017; Gatti et al., 2017; Jin et al., 2020 |
| D4 *mobility* | Schweizer et al., 2013; Spiers & Maguire, 2006; Verdiere et al., 2018; Walter et al., 2001 |
| D5 *self-care* | Small et al., 2001 |
| D9 *community, social and civic life* | Balardin et al., 2017; Bengtsson & Ullén, 2006; Klasen et al., 2012; Mathiak et al., 2011; Pinho et al., 2014; Wan et al., 2011 |
| **Other classification due to other mediating factors predicting meaning** | **References** |

| Necessary activities which satisfy the basic physiological needs such as eating chocolate. | Small et al., 2001 |
| --- | --- |
| Involved a committed activity in which persons committed a large part of their time. | Balardin et al., 2017; Gatti et al., 2017; Schweizer et al., 2013; Walter et al., 2001; Jin et al., 2020; Pan et al., 2021; Chang et al., 2021; Zhou et al., 2022; Shane et al., 2020; |
| Free activity, what people do in their leisure  time.  E.g. Playing a videogame | Balardin et al., 2017; Blood & Zatorre, 2001; Chen et al., 2017; Karmonik et al., 2016; Klasen et al., 2012; Mathiak et al., 2011; Matsunaga et al., 2014; Menon & Levitin, 2005; Mitterschiffthaler et al., 2007; Moghimi et al., 2012; Pereira et al., 2011; Wan et al., 2011; Chabin et al., 2020; Ara et al., 2020; Talami et al., 2020  Klasen et al., 2012; Mathiak et al., 2011; |
| Contracted activity which included regular work  E.g. Taxi drivers driving a cab in virtual circumstances | Bengtsson & Ullén, 2006; Pinho et al., 2014; Spiers & Maguire, 2006; Verdiere et al., 2018; Marion et al., 2021; Jensen et al., 2020  Spiers & Maguire, 2006 |
| Involved the four domains of the classification, due to the imaginary aspect of the activity | Boissoneault et al., 2018; Perreau-Linck et al., 2007; Lee et al., 2020 |
| **5. Degree of meaning in the activities** | **Reference** |
| Activities that had more potential to be meaningful since the activity was clearly linked to the interests, personal characteristics, and life history of the participants | Bengtsson & Ullén, 2006; Chen et al., 2017; Klasen et al., 2012; Mathiak et al., 2011; Small et al., 2001; Spiers & Maguire, 2006; Verdiere et al., 2018; Walter et al., 2001; Wan et al., 2011; Ara et al., 2020; Marion et al., 2020; Chang et al., 2021; Pan et al., 2021; Talami et al., 2020; Shane et al., 2020; Jensen et al., 2020; Zhou et al., 2022; Gatti et al., 2017; Schweizer et al., 2013; Karmonik et al., 2016; Matsunaga et al., 2014; |
| Engaged in an activity that had a meaningful aspect | Balardin et al., 2017; Blood & Zatorre, 2001; Boissoneault et al., 2018; Moghimi et al., 2012; Pereira et al., 2011; Perreau-Linck et al., 2007; Pinho et al., 2014; Jin et al., 2020: Chabin et al., 2020; Lee et al., 2020; Talami et al., , 2020 |

*Supplementary Table S6: Degree of meaning following attributions of Tierney et al. (2020)*

|  | Enjoyable | Suited to indiv. skills, abilities and preferences | Related to personally relevant goals | Engaging | Related to aspects of identity |
| --- | --- | --- | --- | --- | --- |
| Blood et al., 2001 | x | x | x | x | X |
| Small et al., 2001 | x | x |  |  | X |
| Matsunaga et al., 2014 | x | x | x | X |  |
| Perreau-Linck et al., 2007 | x | x | x | x | X |
| Jin et al., 2020 | x | x | x | x | X |
| Chabin et al., 2020 | x | x | x | x | X |
| Ara et al., 2020 | x | x |  | x | X |
| Marion et al., 2021 | x | x |  | x | X |
| Moghimi et al., 2012 | x | x | x | x | X |
| Balardin et al., 2017 | x | x | x | x | X |
| Verdiere et al., 2018 | x | x |  | x | x |
| Chang et al., 2021 | x | x |  | x | X |
| Pan et al., 2021 |  | x |  | x | x |
| Boisonneault et al., 2018 | x | x | x | x | X |
| Spiers et al., 2006 | x | x |  | x | X |
| Schweizer et al., 2013 |  | x |  | x |  |
| Wan et al., 2011 | x | x |  | x | X |
| Gatti et al., 2017 | x | x |  | x | x |
| Walter et al., 2001 |  | x |  | x |  |
| Karmonik et al., 2016 | x | x | x |  |  |
| Bengtsson et al., 2006 | x | x |  | x | x |
| Klasen et al., 2012 | x | x |  | x | X |
| Mathiak et al., 2011 | x | x |  | x | X |
| Pereira et al., 2011 | x | x | x | x | X |
| Pinho et al., 2014 | x | x | x | x | X |
| Zhou et al., 2022 | x | x |  | x | X |
| Talami et al., 2020 | x | x | x | x | X |
| Shane et al., 2020 | x | x |  | x | x |
| Jensen et al., 2020 | x | x |  | x | x |
| Lee et al., 2020 | x | x | x | x | x |
| Chen et al., 2017 | x | x |  | x | x |

1. [↑](#endnote-ref-1)
